# Supplementary material for: Using alternative SMILES representations to identify novel functional analogues in chemical similarity vector searches
Source: Patterns (N Y). 2023 Oct 30;4(12):100865. doi: 10.1016/j.patter.2023.100865 (PMC10724362; doi:10.1016/j.patter.2023.100865)
Supplement: Document S1. Table S1 and Figures S1–S7 [file mmc1.pdf]

Patterns, Volume *4*

Supplemental information

Using alternative SMILES representations to identify novel functional analogues in chemical similarity vector searches

Clayton W. Kosonocky, Aaron L. Feller, Claus O. Wilke, and Andrew D. Ellington

Table S1. Different canonical SMILES string representations for each query molecule.

Query	Canon Alg.	SMILES
Zidovudine	RDKit Atom 0	Cc1cn(C2CC(N=[N+]=[N-])C(CO)O2)c(=O)[nH]c1=O
	RDKit Atom n	O=c1[nH]c(=O)c(C)cn1C1CC(N=[N+]=[N-])C(CO)O1
	OEChem	CC1=CN(C(=O)NC1=O)C2CC(C(O2)CO)N=[N+]=[N-]
Penicillin	RDKit Atom 0	CC1(C)SC2C(NC(=O)Cc3ccccc3)C(=O)N2C1C(=O)O
	RDKit Atom n	c1ccc(CC(=O)NC2C(=O)N3C2SC(C)(C)C3C(=O)O)cc1
	OEChem	CC1(C(N2C(S1)C(C2=O)NC(=O)CC3=CC=CC=C3)C(=O)O)C
Nirmatrelvir	RDKit Atom 0	CC(C)(C)C(NC(=O)C(F)(F)F)C(=O)N1CC2C(C1C(=O)NC(C#N)CC1CCNC1=O)C2(C)C
	RDKit Atom n	N(C(=O)C1C2C(CN1C(=O)C(NC(=O)C(F)(F)F)C(C)(C)C)C2(C)C)C(C#N)CC1CCNC1=O
	OEChem	CC1(C2C1C(N(C2)C(=O)C(C(C)(C)C)NC(=O)C(F)(F)F)C(=O)NC(CC3CCNC3=O)C#N)C
LSD	RDKit Atom 0	CCN(CC)C(=O)C1C=C2c3cccc4[nH]cc(c34)CC2N(C)C1
	RDKit Atom n	c1ccc2[nH]cc3c2c1C1=CC(C(=O)N(CC)CC)CN(C)C1C3
	OEChem	CCN(CC)C(=O)C1CN(C2CC3=CNC4=CC=CC(=C34)C2=C1)C
Fentanyl	RDKit Atom 0	CCC(=O)N(c1ccccc1)C1CCN(CCc2ccccc2)CC1
	RDKit Atom n	c1(CCN2CCC(N(C(=O)CC)c3ccccc3)CC2)ccccc1
	OEChem	CCC(=O)N(C1CCN(CC1)CCC2=CC=CC=C2)C3=CC=CC=C3
SB-759335-B	RDKit Atom 0	CCn1c(-c2nonc2N)nc2cncc(C(=O)N3CCNCC3)c21
	RDKit Atom n	c1ncc2nc(-c3nonc3N)n(CC)c2c1C(=O)N1CCNCC1
	OEChem	CCN1C2=C(C=NC=C2C(=O)N3CCNCC3)N=C1C4=NON=C4N
BMS-536924	RDKit Atom 0	Cc1cc(N2CCOCC2)cc2[nH]c(-c3c(NCC(O)c4cccc(Cl)c4)cc [nH]c3=O)nc12
	RDKit Atom n	C(Nc1cc[nH]c(=O)c1-c1nc2c(C)cc(N3CCOCC3)cc2[nH]1)C (O)c1cccc(Cl)c1
	OEChem	CC1=CC(=CC2=C1N=C(N2)C3=C(C=CNC3=O)NCC(C4=CC(=CC=C 4)Cl)O)N5CCOCC5
558441-90-0	RDKit Atom 0	NCCCCN(Cc1nc2ccccc2[nH]1)C1CCCc2cccnc21
	RDKit Atom n	c1cccc2[nH]c(CN(CCCCN)C3CCCc4cccnc43)nc12
	OEChem	C1CC(C2=C(C1)C=CC=N2)N(CCCCN)CC3=NC4=CC=CC=C4N3
Fluticasone furoate	RDKit Atom 0	CC1CC2C3CC(F)C4=CC(=O)C=CC4(C)C3(F)C(O)CC2(C)C1(OC (=O)c1ccco1)C(=O)SCF
	RDKit Atom n	o1cccc1C(=O)OC1(C(=O)SCF)C(C)CC2C3CC(F)C4=CC(=O)C= CC4(C)C3(F)C(O)CC21C
	OEChem	CC1CC2C3CC(C4=CC(=O)C=CC4(C3(C(CC2(C1(C(=O)SCF)OC(=O)C5=CC=CO5)C)O)F)C)F

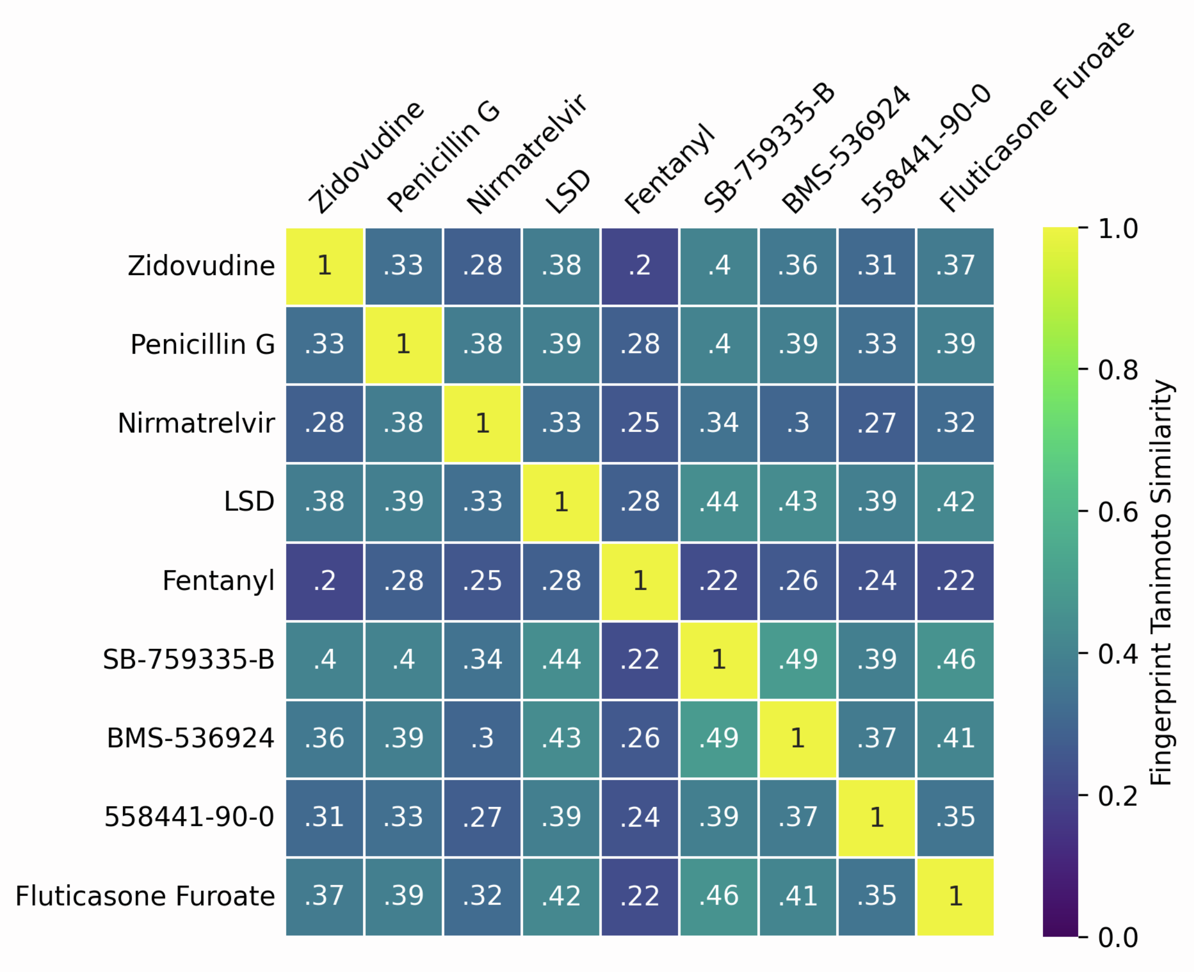

Figure S1: Fingerprint Tanimoto coefficients between each of the query molecules.

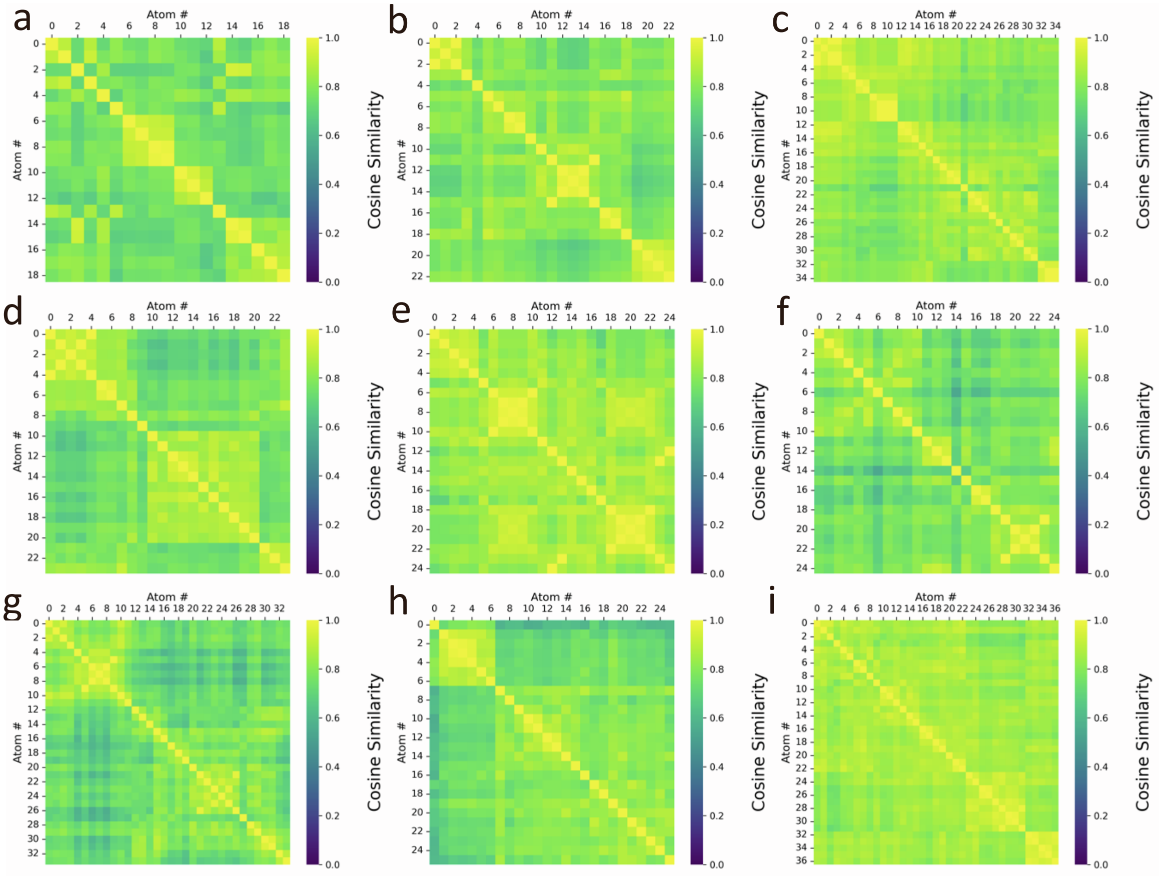

Figure S2: Feature cosine similarity of each RDKit canonicalized query depending on the chosen root atom number. (a-h). In order: zidovudine, penicillin, nirmatrelvir, LSD, fentanyl, SB-759335-B, BMS-536924, 558441-90-0, fluticasone furoate. The canonicalized variant with the lowest feature cosine similarity to the Atom 0 representation was chosen as the “RDKit Atom n” query. The root atoms providing most dissimilar feature vectors were 15 for zidovudine, 13 for penicillin, 21 for nirmatrelvir, 11 for LSD, 17 for fentanyl, 14 for SB-759335-B, 17 for BMS-536924, 10 for 558441-90-0, and 31 for fluticasone furoate.

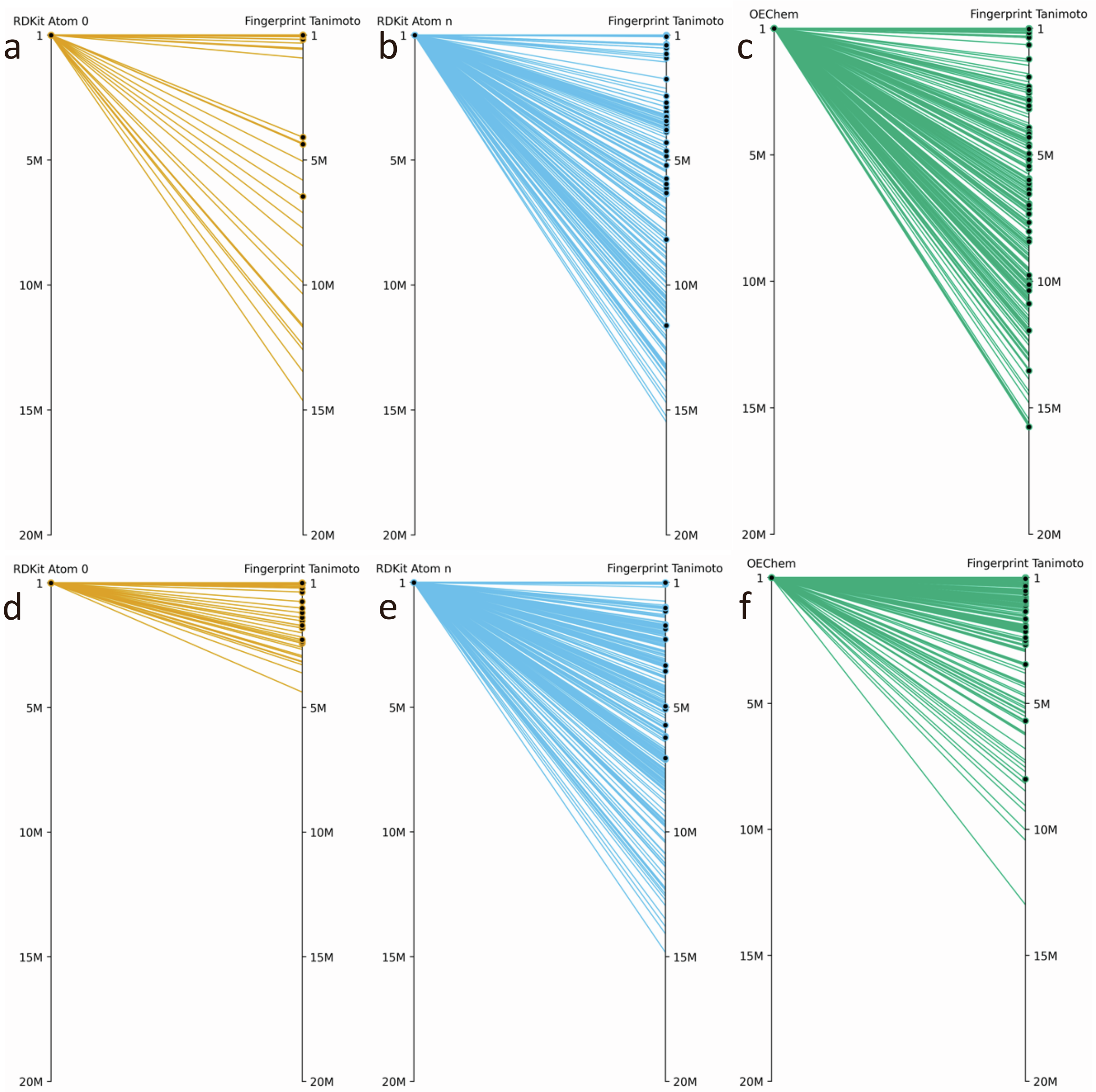

Figure S3. The index rank of each canonicalization's top 250 results for zidovudine and penicillin compared to the index rank that these same molecules scored in a fingerprint Tanimoto search. Black dot indicates molecules functionally similar to the query, as determined by the GPT-assisted patent search. **(a-c)** Zidovudine. **(d-f)** Penicillin.

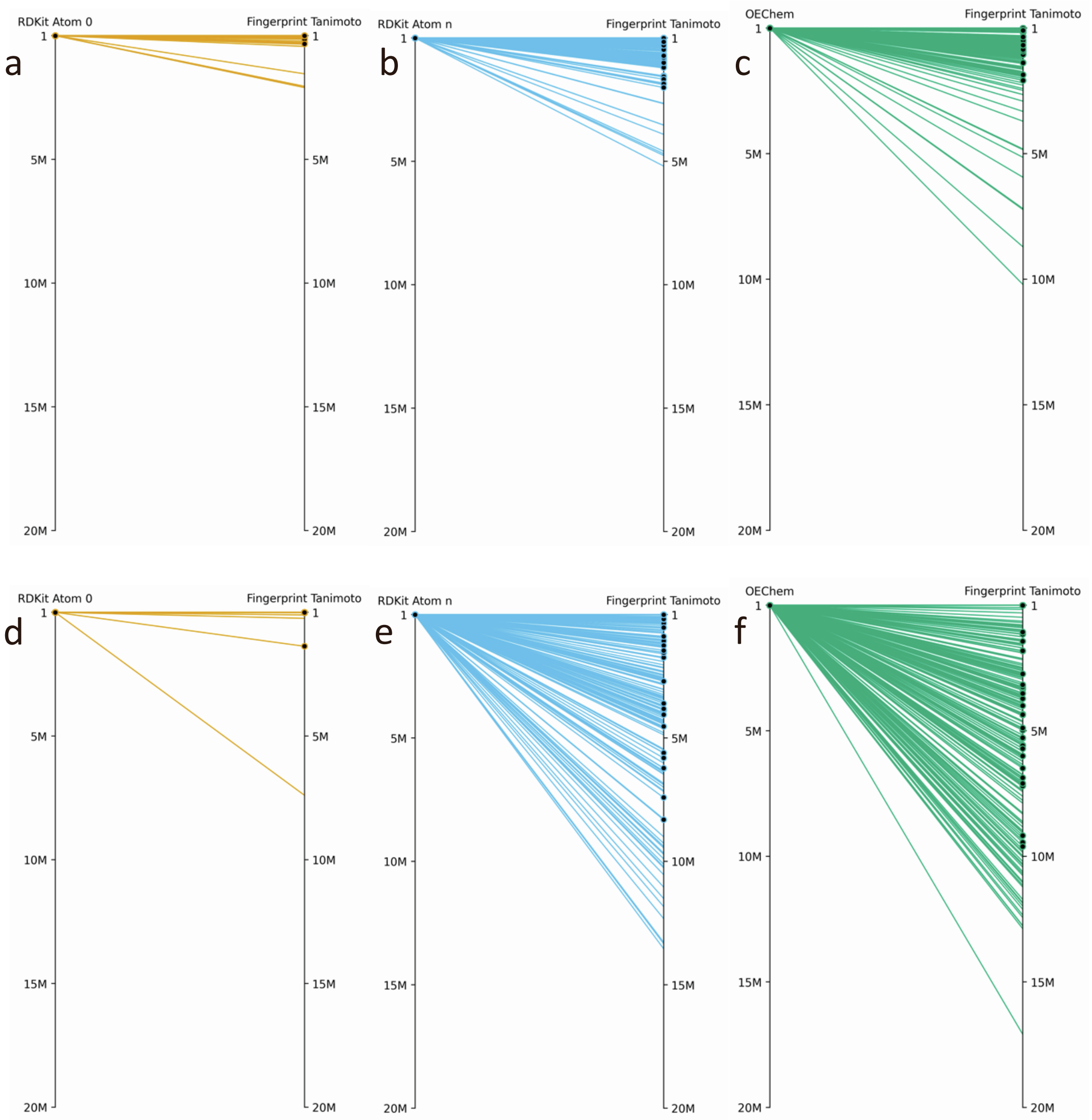

Figure S4. The index rank of each canonicalization's top 250 results for nirmatrelvir and LSD compared to the index rank that these same molecules scored in a fingerprint Tanimoto search. Black dot indicates molecules functionally similar to the query, as determined by the GPT-assisted patent search. **(a-c)** Nirmatrelvir. **(d-f)** LSD.

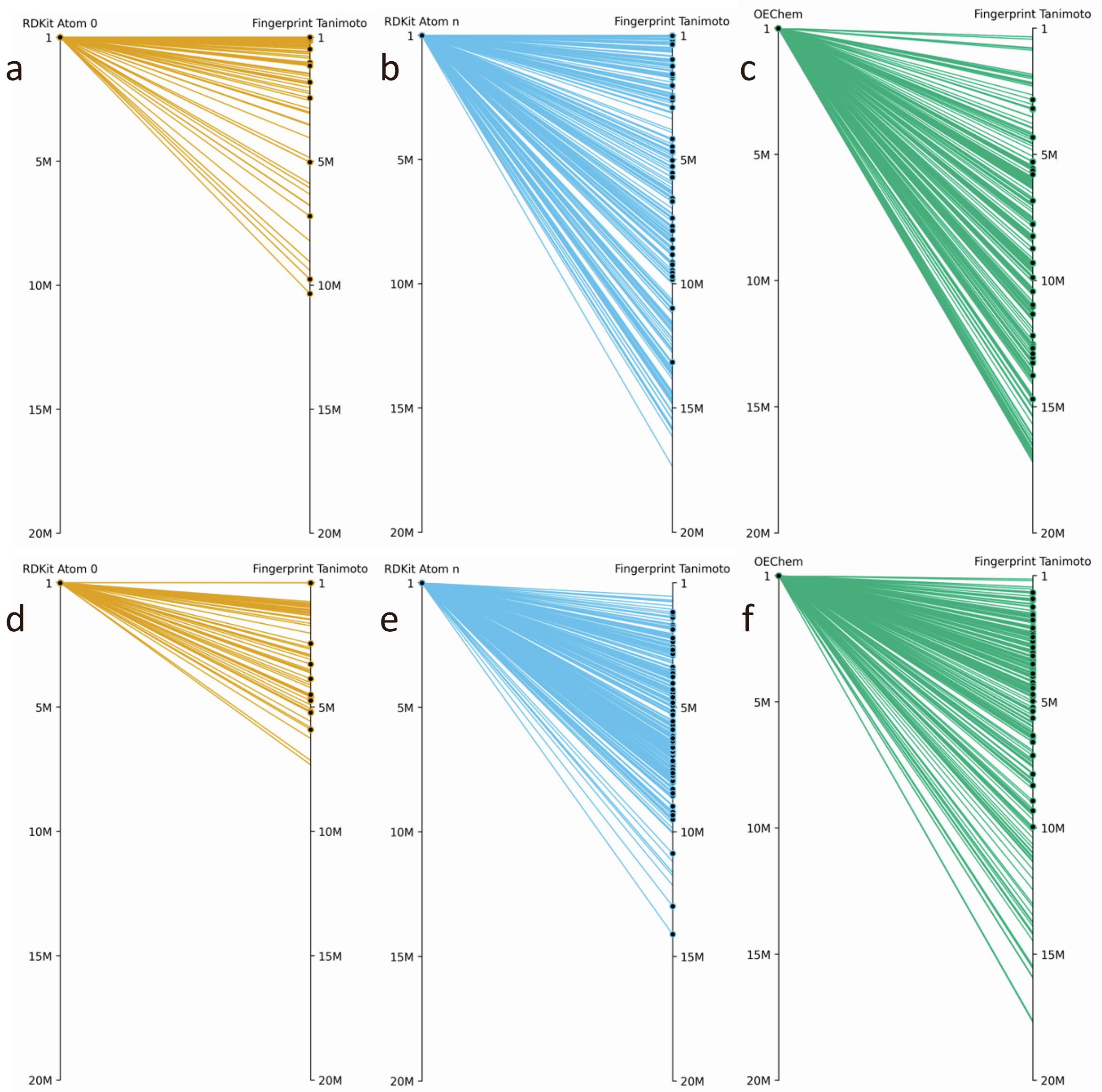

Figure S5. The index rank of each canonicalization's top 250 results for fentanyl and SB-759335-B compared to the index rank that these same molecules scored in a fingerprint Tanimoto search. Black dot indicates molecules functionally similar to the query, as determined by the GPT-assisted patent search. **(a-c)** Fentanyl. **(d-f)** SB-759335-B.

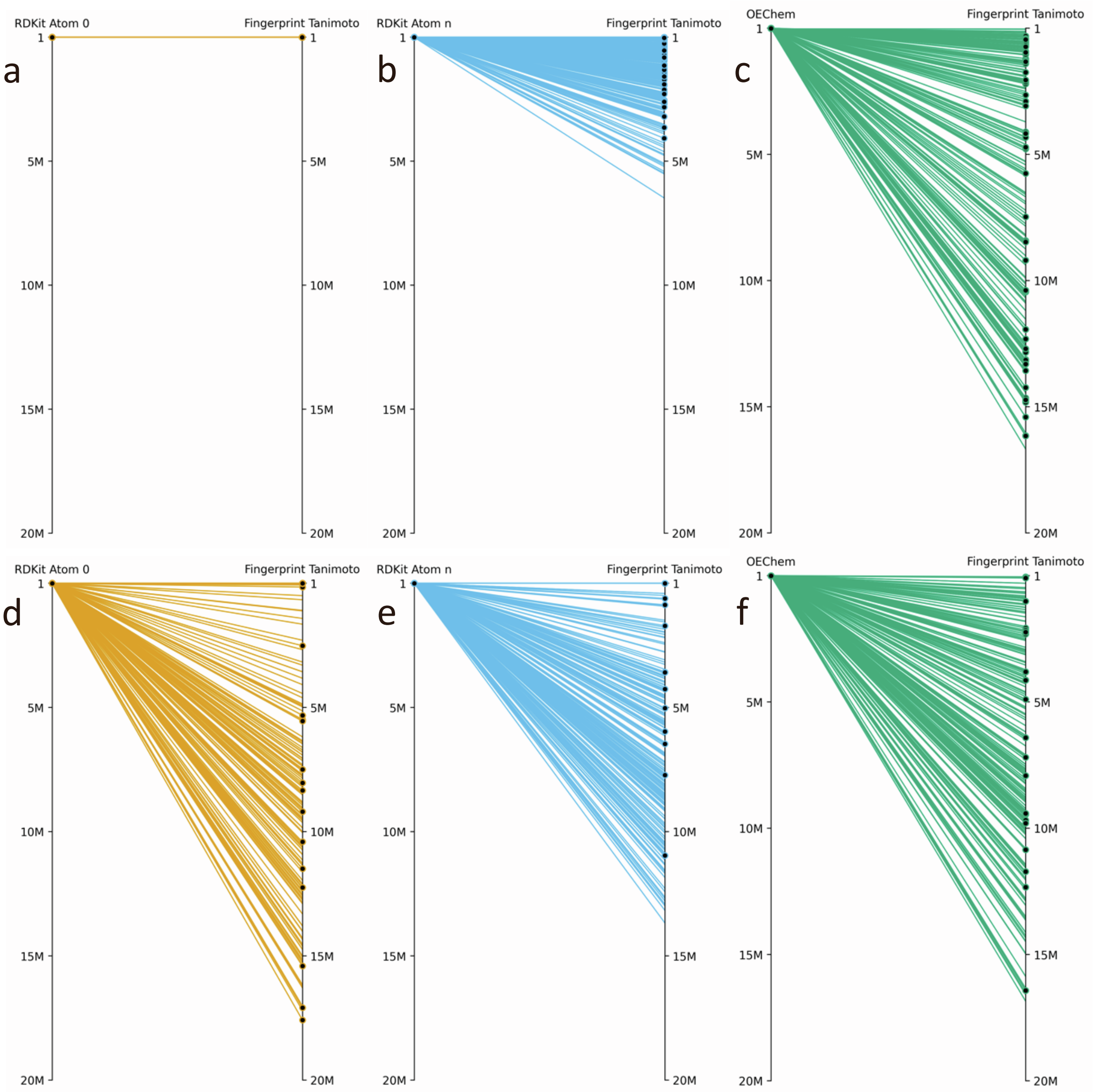

Figure S6. The index rank of each canonicalization's top 250 results for BMS-536924 and 558441-90-0 compared to the index rank that these same molecules scored in a fingerprint Tanimoto search. Black dot indicates molecules functionally similar to the query, as determined by the GPT-assisted patent search. **(a-c)** BMS-536924. **(d-f)** 558441-90-0.

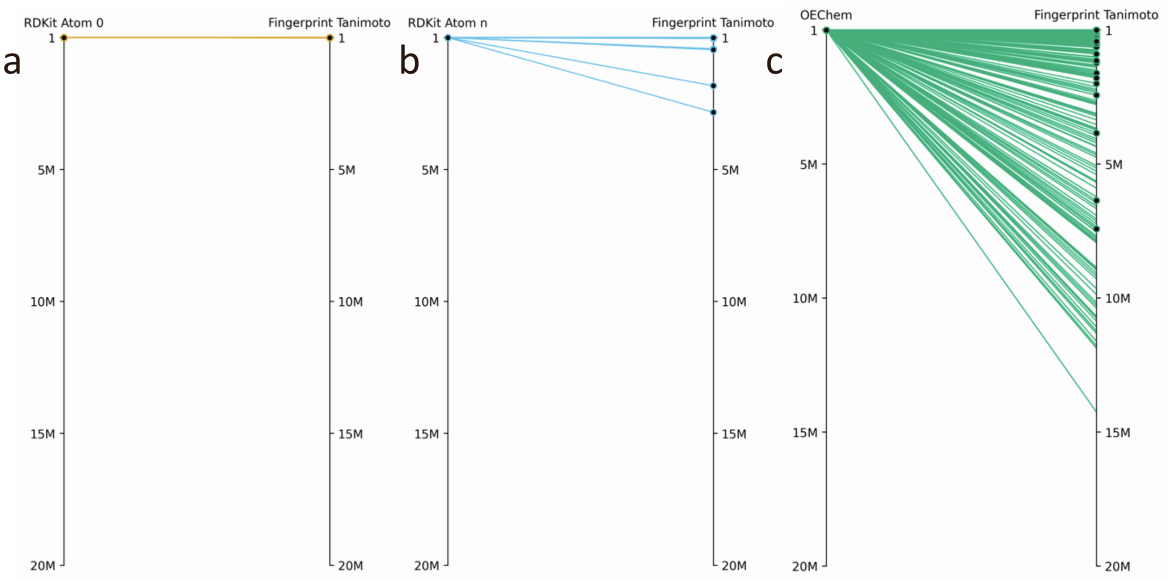

Figure S7. The index rank of each canonicalization's top 250 results for fluticasone furoate compared to the index rank that these same molecules scored in a fingerprint Tanimoto search. Black dot indicates molecules functionally similar to the query, as determined by the GPT-assisted patent search. **(a-c)** Fluticasone furoate.
