# Supplementary material for: Using alternative SMILES representations to identify novel functional analogues in chemical similarity vector searches
Source: Patterns (N Y). 2023 Oct 30;4(12):100865. doi: 10.1016/j.patter.2023.100865 (PMC10724362; doi:10.1016/j.patter.2023.100865)
Supplement: Document S2. Article plus supplemental information [file mmc2.pdf]

Patterns

Article

Using alternative SMILES representations to identify novel functional analogues in chemical similarity vector searches

Graphical abstract

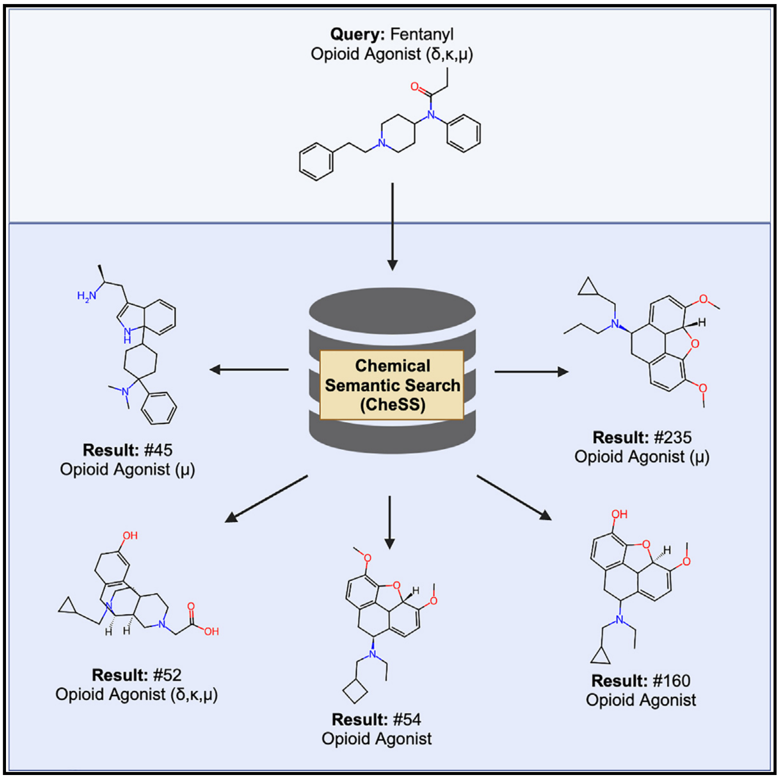

Highlights

- Researchers developed a language model-based chemical similarity search
- The method finds structurally dissimilar molecules with similar function
- Method demonstrated by identifying antivirals, opioids, and serotonin receptor ligands

Authors

Clayton W. Kosonocky, Aaron L. Feller, Claus O. Wilke, Andrew D. Ellington

Correspondence

wilke@austin.utexas.edu

In brief

Researchers developed an approach to chemical similarity search using a chemical language model with SMILES augmentation. Unlike traditional methods, this vector-based search removes memorization of strings and structures, improving the utilization of higher-order token relationships. Testing with diverse queries showed successful identification of molecules with similar patent-derived functionality, even when structures differed significantly. This method may become a valuable tool for discovering new structural classes of molecules with specific target functionalities, benefiting pharmaceutical lead discovery.

Kosonocky et al., 2023, Patterns *4*, 100865
December 8, 2023 © 2023 The Authors.
https://doi.org/10.1016/j.patter.2023.100865

Patterns

CellPress
OPEN ACCESS

Article

Using alternative SMILES representations to identify novel functional analogues in chemical similarity vector searches

Clayton W. Kosonocky,[1] Aaron L. Feller,[1] Claus O. Wilke,[2,4,*] and Andrew D. Ellington[1,3]
[1]Department of Molecular Biosciences, University of Texas at Austin, Austin, TX 78705, USA
[2]Department of Integrative Biology, University of Texas at Austin, Austin, TX 78705, USA
[3]Center for Systems and Synthetic Biology, University of Texas at Austin, Austin, TX 78705, USA
[4]Lead contact
*Correspondence: wilke@austin.utexas.edu
https://doi.org/10.1016/j.patter.2023.100865

THE BIGGER PICTURE Traditional chemical similarity searches have been effective in identifying structurally similar compounds but struggle to find structurally distinct molecules with similar functionality. It would be advantageous to have a similarity search that compares molecules with the functional properties implicit in their structure. Such a search would help rapidly deploy antiviral candidates for future pandemics, combat microbial resistance, and discover better analgesics.

Machine learning models identify complex patterns and present a promising solution to this task. These models learn representations of molecules, encapsulating complex chemical structural knowledge into vector representations. We developed a chemical similarity search using a chemical language model and SMILES-based prompt engineering strategy that identifies functional analogues. This method may be valuable for discovering novel molecules with specific target functionalities in pharmaceutical research.

1 2 3 4 5 **Proof-of-Concept:** Data science output has been formulated, implemented, and tested for one domain/problem

SUMMARY

Chemical similarity searches are a widely used family of *in silico* methods for identifying pharmaceutical leads. These methods historically relied on structure-based comparisons to compute similarity. Here, we use a chemical language model to create a vector-based chemical search. We extend previous implementations by creating a prompt engineering strategy that utilizes two different chemical string representation algorithms: one for the query and the other for the database. We explore this method by reviewing search results from nine queries with diverse targets. We find that the method identifies molecules with similar patent-derived functionality to the query, as determined by our validated LLM-assisted patent summarization pipeline. Further, many of these functionally similar molecules have different structures and scaffolds from the query, making them unlikely to be found with traditional chemical similarity searches. This method may serve as a new tool for the discovery of novel molecular structural classes that achieve target functionality.

INTRODUCTION

Applications for small molecules in modern society are various and widespread, including treatment of heritable disease, pathogen inhibition, and the generation of functional materials for use in electronics and consumer goods. Molecular function emerges from structure, but it is not always obvious how this emerges from first principles due to the dependence of function on the target molecule.[1] Many traditional pharmaceuticals and specialty chemicals are the result of natural product exploration.[2–5] These first-generation molecules act as starting points upon which new molecules are engineered for furthering desired functionality.[6] Structural neighbors often share similar functionality, as the relevant chemistry may be unchanged or improved.[7] However, molecules with low structural similarity can act on the same target despite highly different structure, as is the case with morphine and fentanyl on the mu-opioid receptor.[8]

This is an open access article under the CC BY license (http://creativecommons.org/licenses/by/4.0/).

There are numerous contemporary approaches applying machine learning to chemistry.[9–16] In particular, the application of language models to this space has led to surprising success in predicting biochemical features such as drug-likeness and protein-ligand interactions.[11,13] These methods require string representations of molecules, commonly using the simplified molecular-input line-entry system (SMILES).[17] Language models are often trained in a self-supervised manner through masked language modeling, in which the objective function is tied to sequence reconstruction (i.e., feeding the model a masked or partial input with the goal of reconstructing the original sequence). It was recently demonstrated that a chemical language model, although trained only on SMILES strings, correctly predicted complex biophysical and quantum-chemical properties.[18] This points to the possibility that these chemical language models develop a latent space that allows for the emergence of higher-order biochemical comprehension.

Recently, computationally generated chemical libraries have grown to surpass 37 billion commercially available compounds.[19] This growth spurred the pharmaceutical industry toward the practice of computationally pre-screening chemicals for resource-efficient discovery in the laboratory.[20] One primary class of computational pre-screening methods are chemical similarity searches. These methods have historically used structure-based comparisons, notably 2D/3D pharmacophore searches and the fingerprint Tanimoto search, the latter of which computes a hierarchical list of molecules ranked by molecular substructure similarity to a given query.[21,22]

Chemical language models (CLMs) have previously been applied to drug discovery, in particular *de novo* molecule generation and chemical similarity searches. *De novo* methods autoregressively generate novel molecular strings using recurrent neural networks, transformers, or generative pre-trained transformer (GPT) models, typically after fine-tuning toward a specific molecular dataset or downstream task to narrow the solution space.[14–16,18] *De novo* molecule generation has shown promise but is currently limited due to a lack of generalizability and guaranteed synthesizability.[23] In contrast, a CLM-based chemical similarity search has the advantage of computational speed, generalizability, and high database control to ensure synthesizability. Sellner et al. recently created a novel transformer-based chemical similarity search to approximate previous structure-based methods, emphasizing the computational efficiency of a vector search that surpassed that of traditional approaches.[12] This method was trained to identify molecules with high structural similarity to the query molecule, but it is often the case that we wish to find structurally dissimilar molecules that retain their target functionality. These structurally distant molecules are beneficial in combatting antibiotic resistance, reducing drug side effects, and improving our understanding of chemistry. Such a CLM-based search does not currently exist.

Here, we describe a CLM-based chemical similarity search that identifies structurally dissimilar molecules with similar patent-derived function to a given query molecule. This method computes CLM embedding similarities between a query SMILES string and a chemical database. Keeping the SMILES canonicalization algorithm constant between the query and database results in a vector search that approximates recent transformer-based chemical similarity search methods.[12] However, when the query SMILES string is canonicalized with a different algorithm than was used for the database, the reliance on structural similarity is diminished while functional similarity is retained. This behavior seemed reasonable given the previous literature on SMILES augmentation for model training and leads to the hypothesis that we are performing a partially out-of-distribution query that confounds the model enough to eliminate memorization-based structural comparisons but not enough to impede semantic understanding.[24,25] This method fundamentally differs from existing literature in that SMILES augmentation is employed during inference of a chemical language model rather than during model training. This method was tested across three canonicalizations and nine query molecules, ultimately showing that queries canonicalized differently than the database were able to identify structurally distinct functional analogues.

RESULTS

In silico drug discovery methods have long relied on chemical similarity searches in pharmaceutical pipelines.[22] Recently, language models have been successful in biochemical prediction tasks for chemical properties such as drug-likeness, protein-ligand interactions, and other metrics.[11,13] As CLMs have been used for prediction of a multitude of chemical characteristics, it is plausible that embeddings from these models are structured in a way that approximates a summary of molecular properties for a given molecule. SMILES representations can be used to represent chemical structures as strings and can be used as inputs to CLMs, but, due to the nature of chemical connectivity, there are often many valid representations for the same molecule.[17] This multiplicity of input formats results in the CLM generating different embeddings for the same molecule, which can either be mitigated through string standardization (known as canonicalization) or utilized as a data augmentation technique to create a SMILES representation-invariant CLM.[24,25] The latter is predominantly used during model training, reducing overfitting to particular strings by teaching the model that a given structure has multiple corresponding string representations.[24,25]

However, SMILES augmentation during model inference, rather than model training, may prove to be advantageous for a chemical similarity search. Chemical databases often include structural permutations of well-studied chemicals, causing embedding-based similarity searches to be dominated by low-level structure-based comparisons. SMILES strings representing the same structure often share characters and substrings with one another, the primary difference being the specific canonicalization rules used to assemble the string for a given molecule. For CLMs trained on one canonicalization, inputs using unseen canonicalizations would be out of distribution, not on the token identity level but on the immediate token relationship level for a given molecule. This is because the underlying tokens and token relationships are not entirely unknown to the model but are novel in the context of the given molecule. It seems plausible that a vector comparison between embeddings from two different canonicalizations could ignore the differences in low-level string and structural data and instead use whole-molecule properties approximating molecular function, thus serving as a novel prompt engineering strategy for the discovery of structurally distinct functional analogues. To our knowledge, this behavior has been largely unexplored.

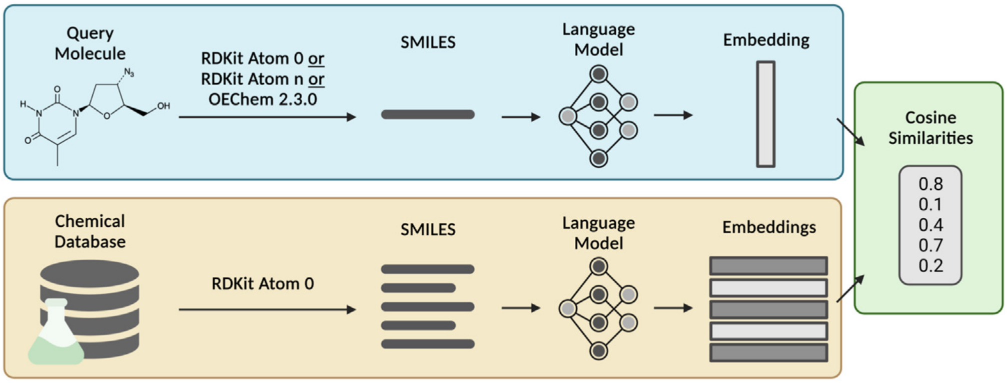

Figure 1. Chemical semantic search

The query molecule and chemical database are converted into SMILES strings, canonicalized, and then inputted into a language model to obtain embeddings. The cosine similarity between the query embedding and database embeddings is computed, resulting in a vector of embedding similarities. The database is canonicalized with RDKit Atom 0, whereas the queries are canonicalized using one of the following: RDKit Atom 0, RDKit Atom n, or OEChem 2.3.0. Figure created with BioRender.com.

To test this hypothesis, we built a CLM-embedding-based chemical similarity search utilizing one canonicalization for the CLM and database and another canonicalization for the query. A pipeline was developed to perform a chemical similarity search using cosine similarity on CLM embeddings obtained from ChemBERTa, a self-supervised transformer-based encoder model.[26] This pipeline was named the Chemical Semantic Search (CheSS) and is outlined in Figure 1. ChemBERTa was pretrained on SMILES strings canonicalized using the default RDKit implementation, herein referred to as "RDKit Atom 0."[26,27] We converted the SureChEMBL dataset into a molecular database of 18.9 million patent-associated RDKit Atom 0 SMILES strings, upon which CheSS searches were performed. We then explored how the CheSS search results were affected by three different query SMILES canonicalizations. The first was RDKit's default canonicalization RDKit Atom 0 (Tables 1 and S1). The second canonicalization was created by varying the RDKit root atom number to create a string that resulted in maximum distance in feature space from the RDKit Atom 0 query, herein referred to as "RDKit Atom n" (Tables 1 and S1; Figure S2). The third used OEChem 2.3.0, a markedly different algorithm, to canonicalize the query, referred to as "OEChem" (Tables 1 and S1). CheSS searches utilizing these three canonicalizations differed only in the representation of the query molecule with the database and model remaining constant.

A CheSS search using each of the three query canonicalizations was conducted on nine structurally and functionally distinct molecules: zidovudine, penicillin, nirmatrelvir, lysergic acid diethylamide (LSD), fentanyl, SB-759335-B, BMS-536924, 558441-90-0, and fluticasone furoate (Figures 2 and S1).

Table 1. SMILES string differences for zidovudine based on canonicalization

Query	Canonicalization algorithm	SMILES
Zidovudine	RDKit Atom 0	Cc1cn(C2CC(N=[N+]=[N−])C(CO)O2)c(=O)[nH]c1=O
	RDKit Atom n	O=c1[nH]c(=O)c(C)cn1C1CC(N=[N+]=[N−])C(CO)O1
	OEChem	CC1=CN(C(=O)NC1=O)C2CC(C(O2)CO)N=[N+]=[N−]

Unabridged SMILES for each query are listed in Table S1.

Statistical analysis of canonicalized query representations

For each query molecule, several pairwise similarity metrics were calculated between the three canonicalizations (Figure 3). Gestalt pattern matching, a string similarity metric, showed that each query canonicalized into different strings with a mean pairwise value of 0.47 across canonicalizations (n = 9) (Figure 3). Because the CLM does not directly receive strings as inputs but instead receives the tokenized representations (integer-mapped subsections) of strings, the token vectors were analyzed to understand how these strings would be presented to the model. The ratio of shared tokens indicated that the query strings were converted using different input tokens (mean pairwise ratio of 0.67 across canonicalizations) (Figure 3). Similarly, token vectors had variable length depending on canonicalization, with some queries differing by a factor of nearly 2 (Figure 3). Token vectors directly affect featurization, or the model's interpretation of the input. As hypothesized, different canonicalizations generated distinct embeddings (mean pairwise embedding cosine similarity of 0.64), indicating that the model interpreted different canonicalizations of the same molecule as quite distinct inputs (Figure 3).

String, token, and structural relevance of top search results

To explore how different canonicalizations affect vector search behavior, similarity metrics were obtained comparing each canonicalized query to its respective top 250 CheSS search results. Queries canonicalized with RDKit Atom 0 yielded compounds high in structural similarity, as measured by the fingerprint Tanimoto coefficient (a metric of molecular substructure similarity), which had a mean coefficient of 0.67 (n = 2,250) (Figure 4A). In contrast, the mean fingerprint Tanimoto coefficients for RDKit Atom n and OEChem were 0.45 and 0.40, respectively. Similarly, the Murcko scaffold fingerprint coefficient followed this trend with a mean scaffold similarity of 0.64, 0.33, and 0.30 for RDKit Atom 0, RDKit Atom n, and OEChem. For RDKit Atom

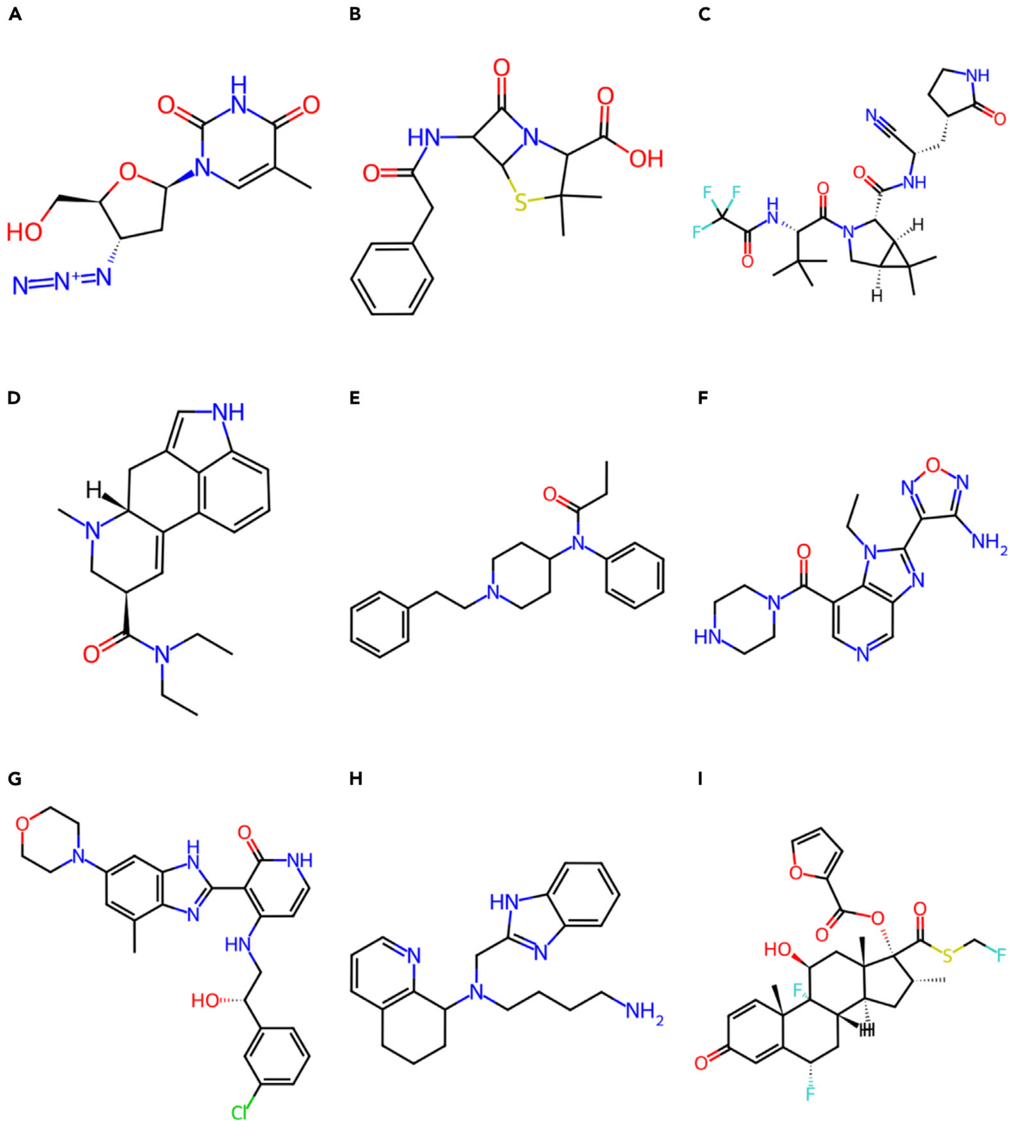

Figure 2. Structures of query molecules
All queries were made achiral during canonicalization.
(A) Zidovudine.
(B) Penicillin.
(C) Nirmatrelvir.
(D) LSD.
(E) Fentanyl.
(F) SB-759335-B.
(G) BMS-536924.
(H) 558441-90-0.
(I) Fluticasone furoate.

0 canonicalized queries, 27.5% of the top 250 results would be identified using a fingerprint Tanimoto search with a cutoff as high as 0.80, indicating that nearly a quarter of the results were 1–2 atomic changes aways from the query molecule (Figures 4A and 4B). In contrast, only 2.8% and 1.8% of the top results for RDKit Atom n and OEChem would have been found from this same high-cutoff fingerprint search. This indicates a significant structural divergence in results when using alternative query canonicalizations (Figures 4A–4D).

It was found that RDKit Atom 0 canonicalized queries returned molecules with a high number of shared tokens to the query token vector (mean ratio of 0.74), whereas this was not the case with RDKit Atom n and OEChem (means of 0.57 and 0.58). This indicates that the model's ability to memorize tokens to determine embedding similarity was reduced by querying with different canonicalizations (Figure 4A). Interestingly, it was observed that the token vector length ratios for all canonicalizations' results had mean values around 1.0, indicating that token

CellPress
OPEN ACCESS

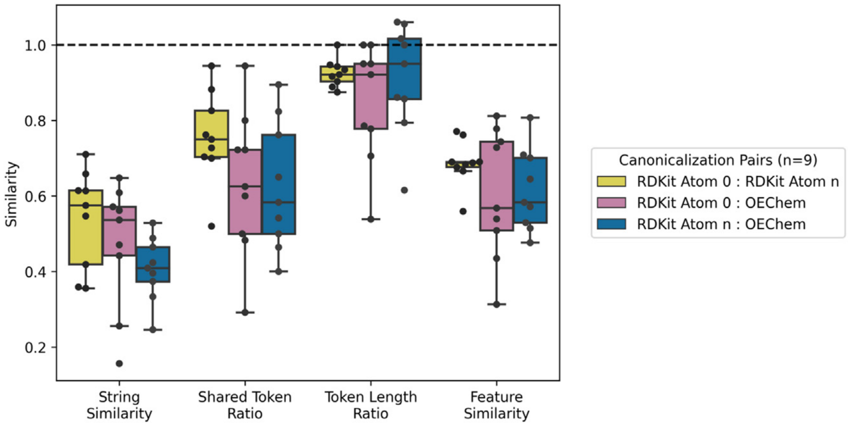

Figure 3. Similarity metrics between the three canonicalized representations of each query molecule

String similarity, as measured with gestalt pattern matching, demonstrates that different canonicalizations result in markedly different strings. Shared token ratio and token length ratio indicate that these strings were tokenized into different inputs to the CLM. Feature similarity demonstrates that the differently canonicalized queries' token vectors were interpreted differently by the model resulting in increased spread across feature space. Feature similarity was determined by cosine similarity between ChemBERTa vector embeddings. Deviations from 1.0 for each metric represent divergence between canonicalized queries.

vector length heavily influences feature space location and thus similarity (Figure 4A). This means that token vector length may constrain CLM-based similarity searches to confined regions of chemical space, with alternative canonicalizations acting as ways to bypass this predominant search criteria through variations in token vector length, thereby allowing exploration of distant regions of chemical space to create more comprehensive and far-reaching similarity searches (Figures 4C and 4D).

Large language model-assisted patent search reveals functional analogues

The data discussed thus far demonstrates that alternatively canonicalized queries find structurally distinct compounds but does not confirm whether the returned molecules are functionally relevant to the query. Patent literature represents a rich source of information on molecular functionality. Summarizing patents into brief and relevant functional descriptors is a tedious task that would take an intractable amount of time over the 9,750 CheSS search results' 17,980 unique associated patents. This limitation was bypassed with the creation of an automated large language model (LLM)-assisted patent summarization and functional determination pipeline.

This workflow takes a SMILES string, finds PubChem compound IDs (CIDs) with the same connectivity, obtains linked patent IDs (limited to 10 per molecule), and scrapes Google Scholar to obtain the patent title, abstract, and description (Figure 5). This information is then combined with a prompt and passed into GPT-3.5-turbo to obtain a set of brief one- to three-word functional descriptors (Figure 5). The descriptors are aggregated per molecule and combined into a second prompt alongside pre-defined descriptors of the relevant query molecule's functionality, which is then passed into GPT-4 to determine whether the two molecules have similar functionality (Figure 5). This method was manually validated for performance on a random 100 molecules from the 9,750 search results, analyzing both label accuracy per patent (n = 1,028 labels) and functional similarity to the relevant query (n = 100 molecules) (Tables 2, 3, and 4). The results of this validation demonstrated 95% accuracy, 94% precision, and 96% recall on functional similarity determination, with the limiting factor being that some of the molecules had been linked to patents in which they served as intermediates rather than the patent product itself.

Functional similarities of the top 250 results for their respective nine query molecules were analyzed (Figure 6). Seventy-five percent of the RDKit Atom 0 results were functionally similar to their query, 42% for RDKit Atom n, and 27% for OEChem. However, the alternative canonicalizations returned molecules that contained significantly dissimilar structures and scaffolds from the query, with mean whole-molecule fingerprint Tanimoto similarities of 0.72, 0.54, and 0.51 for RDKit Atom 0, RDKit Atom n, and OEChem, respectively, and mean scaffold fingerprint Tanimoto similarities of 0.69, 0.43, and 0.41 (Figure 5).

Upon closer inspection of the structurally dissimilar functional analogues from the alternatively canonicalized CheSS queries, it was found that many of these molecules represented structural classes distinct from the query. These are illustrated with the nirmatrelvir, LSD, and fentanyl OEChem-canonicalized searches (Figure 7). Nirmatrelvir, a peptidomimetic severe acute respiratory syndrome coronavirus 2 (SARS-CoV-2) main protease inhibitor, returned a wide range of non-peptidomimetic protease and peptidase inhibitors (Figure 7A). LSD, an ergoline 5-HT2A agonist, returned many non-ergoline 5-hydroxytryptamine (5-HT, serotonin) receptor ligands not obviously derivable from LSD (Figure 7B). Fentanyl, an elongated piperidine opioid agonist, returned several morphinan and non-fentanyl opiates (Figure 7C).

One artifact of determining functional similarity from patent literature is that the true positive rate for functionally relevant molecules may be higher than the value obtained in this study, as the relevant assay is not guaranteed to have been conducted and reported for a given molecule. As a consequence, the molecules deemed to have irrelevant functionality are curiously the most desirable leads for novel drug discovery and repurposing. Taken together, these hits provide support for the hypothesis that changing query canonicalization for a transformer-based chemical similarity search can lead to the discovery of structurally novel, yet functionally relevant, chemical compounds.

DISCUSSION

Explanation of search behavior

We find that alternative canonicalizations influence CheSS behavior through changes in the token vectors, which appear to cause higher-order token relationships to dominate in the embeddings. There are stark differences between RDKit and OEChem

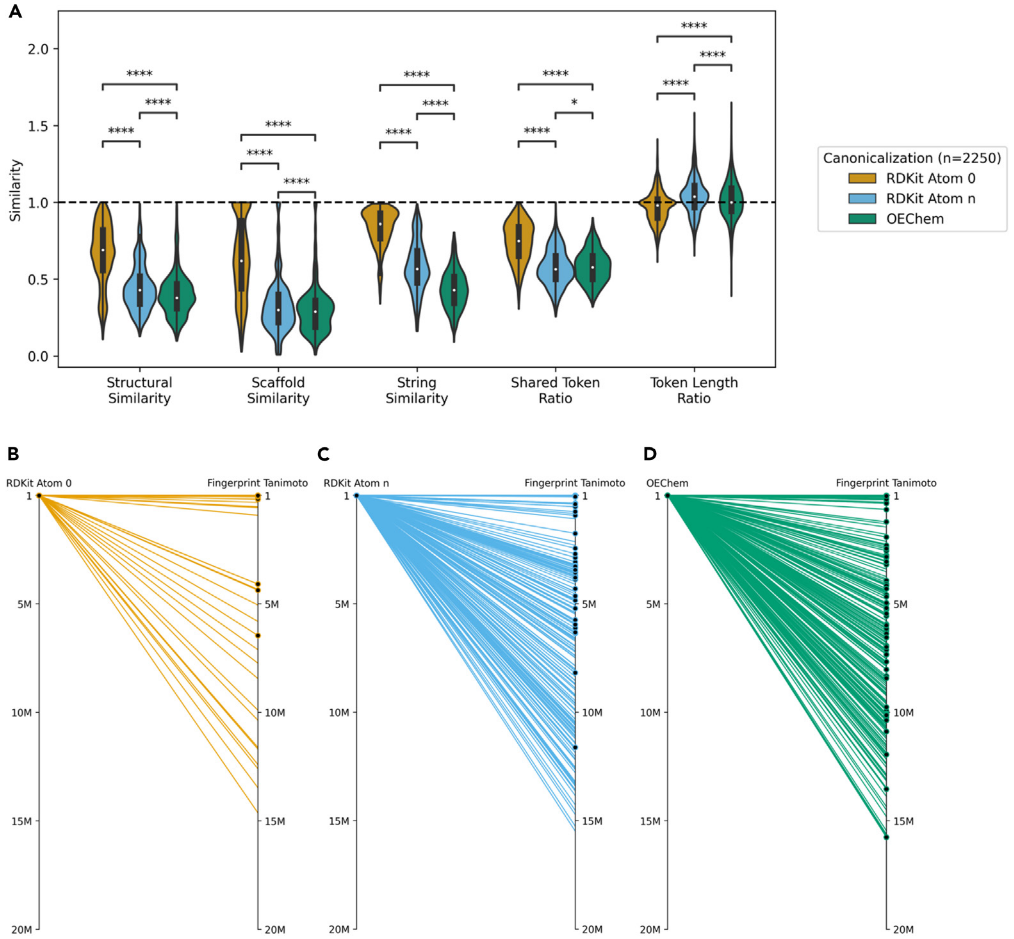

Figure 4. Search behavior depends on canonicalization

(A) Similarity metrics for all CheSS searches between each canonicalized query and its respective top 250 results. Compared to consistent canonicalization, the top 250 results from alternative canonicalizations were significantly more dissimilar in structural, scaffold, string, and shared token similarity. Structural similarity measured by whole-molecule fingerprint Tanimoto similarity, scaffold similarity measured by scaffold fingerprint Tanimoto similarity, string similarity measured by gestalt pattern matching. Asterisks indicate the level of statistical significance for two-sided independent t tests (ns, $p < 1.0$; *$p < 0.05$; **$p < 0.01$; ***$p < 0.001$; ****$p < 0.0001$).

(B–D) The index rank of each canonicalization's top 250 results for zidovudine compared to the index rank that these same molecules scored in a fingerprint Tanimoto search. Black dot indicates molecules functionally similar to the query, as determined by the LLM-assisted patent search. Rank plots for all queries are listed in Figure S7.

canonicalizations, notably their differences in the representation of aromatic rings. For conjugated structures, OEChem prefers the Kekulé form (C1 = CC = CC = C1), while RDKit prefers to use lowercase with assumed aromaticity (c1ccccc1). These differences, among others, result in markedly different tokenization, both in the composition and the length of the tokenized vectors (Figure 3).

SMILES strings generated from alternative canonicalizations can be viewed as out-of-distribution queries, not in the token identities (as no new tokens were created by changing canonicalization) but in the low-level token relationships for a given molecule. This relaxation of embedding the immediate token relationships can be seen in the diminished shared token ratios of alternatively canonicalized queries' results as low-level pattern matching can no longer occur (Figure 4A). However, higher-order token relationships are still maintained across all searches regardless of canonicalization, as demonstrated by the constraint on token vector length (Figure 4A). It is the other opaque high-order token relationships that we believe to be causing the language model to embed functionally similar molecules, independent of structure, close together in feature space when alternative canonicalizations are used (Figure 5).

When CLMs are forced to go beyond simple token patterns to determine similarity, more nuanced relationships take hold. Given the current understanding of transformers, it is indiscernible what these relationships are, but, based on our analysis, we find it possible that the CLM may, for example, key in on the apposition of functional groups in space similar to how receptors perceive ligands.

Drawbacks, future improvements, and potential for misuse

Molecules with a small number of atoms differ only minimally in their canonicalized representations. This leads to homogeneity between query representations, reducing the applicability of

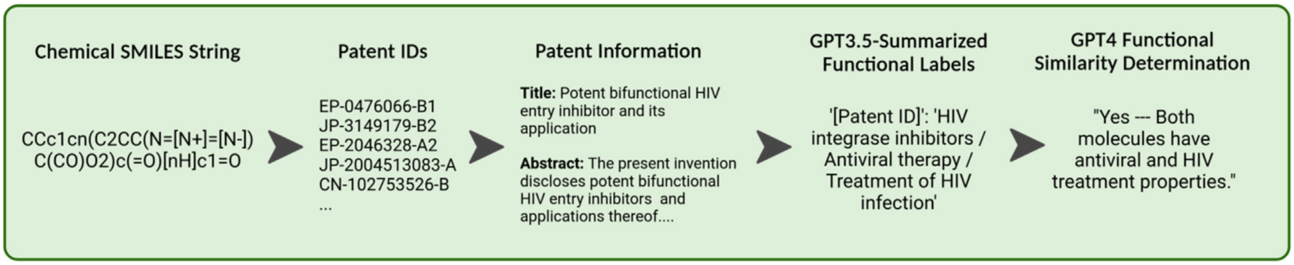

Figure 5. Patent summarization and functional determination pipeline

Chemical SMILES strings are converted to PubChem CIDs based on same connectivity, then associated patent IDs are obtained. These are then used to obtain the patent title, abstract, and description from Google Scholar. The patent information is then passed into GPT-3.5-turbo with a prompt to obtain summarized functional labels. These labels are then passed into GPT-4 with pre-defined labels to determine functional similarity between the query and molecule in question. Figure created with BioRender.com.

CheSS. The top results for OEChem-canonicalized queries with a high number of double bonds included many porphyrin and phthalocyanine derivatives, even though they were functionally irrelevant to the query. The likely reason for this is that the query contained many double-bond characters, which resulted in unusually long vectors during tokenization. This caused the model to interpret the query similarly to the large conjugated molecules that RDKit canonicalized using explicit double bonds rather than assumed aromaticity. This behavior could be avoided through pruning the database or results of large conjugated molecules.

The language model used herein, ChemBERTa, has been outperformed by more modern language models such as MolFormerXL.[18,26] CheSS could be improved by utilizing these state-of-the-art models to generate a richer feature space, presumably allowing for better similarity searches. Further, the models discussed herein are self-supervised, resulting in broad functional similarity comparisons. The use of fine-tuned models to generate the embeddings would result in more specific functional comparisons, examples being implicit comparisons of lipophilicity, specific receptor binding, and drug-likeness. Further, the similarity metric used to compare embeddings herein was cosine similarity, but Euclidean distance would be an equally valid, or potentially superior, metric. The CheSS framework is extensible to any dataset, CLM, SMILES representation, and similarity metric, and we invite discussions toward optimal configurations.

We also note the threat of dual use for chemical machine learning models.[49] While the model used in our implementation of CheSS was self-supervised and has not been trained for identifying toxic molecules, any successful chemical similarity search tool carries inherent risks. We therefore advise caution in considering public implementations of these tools and recommend restricting searches to avoid queries with the potential for malicious use.

Conclusions

In this study, we created a chemical similarity search utilizing a transformer-based encoder chemical language model to generate embeddings upon which similarity scores can be computed. From this, we designed a query strategy that expands upon the notion of chemical semantic searches by creating a method able to identify structurally dissimilar molecules with similar function. We demonstrate the success of this method through the identification of compounds that bind the same receptor, or family of receptors, as that of the query molecule despite significant structural dissimilarity. This method may aid in drug repurposing efforts and the discovery of new structural classes of molecules with desired function. In addition to the chemical similarity search, we developed a LLM-assisted patent summarization and functional similarity determination pipeline that has been shown to determine molecular functionality with very high accuracy. We believe that CheSS and the canonicalization-based prompt engineering method discussed herein will be of broad interest to the chemical community, as it provides an example for how SMILES-based prompt engineering can modulate chemical language model embeddings to discover new molecules with desired function.

EXPERIMENTAL PROCEDURES

Resource availability

Lead contact

Further information should be directed to and will be fulfilled by the lead contact, Claus Wilke (wilke@austin.utexas.edu).

Table 2. Validation for LLM-assisted patent summarization

	Label relevant to patent	Label refers to MOI, target of MOI, or downstream effects of MOI	Label refers to MOI, target of MOI, downstream effects of MOI, or molecules of which MOI is an intermediate
Patent summarization (n = 1,028)	1.00	0.81	0.94

Achiral SMILES for the MOI were converted to same-connectivity PubChem CIDs, and associated patents were retrieved and fed into GPT-3.5-turbo for summarization into brief labels. Manual validation was performed on 100 molecules randomly selected from the 27 CheSS searches' top 250 results.

Table 3. Validation for LLM-assisted functional similarity

	Accuracy	Precision	Recall	Correct reasoning
Functional similarity (n = 100)	0.95	0.94	0.96	0.91

Patent summarization labels were fed into GPT-4 alongside labels for the query, and the model was prompted to determine if the two sets of labels describe molecules with similar functionality. Manual validation was performed on 100 molecules randomly selected from the 27 CheSS searches' top 250 results.

Materials availability

This study did not generate new unique materials.

Data and code availability

The top 250 results from each query, as well as the project code used to generate search results, have been deposited at Zenodo[50] and are publicly available as of the date of publication. The code is also available at https://github.com/kosonocky/CheSS. This paper utilizes the existing, publicly available, SureChEMBL database.[51] Any additional information required to reanalyze the data reported in this paper is available from the lead contact upon request.

CheSS overview

CheSS is a molecular search framework that uses language model-encoded embeddings to compute similarity scores across molecular space. A database of molecules is encoded as strings using the SMILES format.[17] A chemical language model is used to generate an embedding for each molecule in the database as well as the query molecule. The cosine similarity between the query vector and each database vector is computed, resulting in a vector of embedding similarities.

Language model

ChemBERTa was used as the language model to generate embeddings.[26] This was a Bidirectional Encoder Representations from Transformers (BERT) model with 12 hidden layers of size 768, and was trained on 10 million random non-redundant achiral SMILES strings selected from PubChem.[52] ChemBERTa was chosen over newer, higher-parameter BERT models due to the ease of implementation and publicly available dataset. ChemBERTa does not support isomeric SMILES (chirality), and all SMILES were canonicalized before input.

Table 4. Functional descriptors of query molecules

Molecule	Descriptors
Zidovudine	reverse transcriptase inhibitor,[28] antiviral,[29] albumin ligand,[30] phosphorylase ligand[31]
Penicillin	antibiotic,[3] antibacterial,[3] beta-lactamase ligand,[32] penicillin-binding protein ligand[3]
Nirmatrelvir	SARS-CoV-2 inhibitor,[33] antiviral,[33] protease inhibitor,[33] peptidase inhibitor[33]
LSD	dopaminergic,[34] serotonergic,[35] histamine receptor ligand,[36] psychoactive[34,35,37]
Fentanyl	opioid receptor ligand (mu, kappa, delta),[38,39] analgesic,[38] anesthetic[38]
SB-759335-B	serine-threonine kinase inhibitor,[40] kinase inhibitor,[40,41] MSK inhibitor,[41] AKT inhibitor,[40,41] YES inhibitor[42]
BMS-536924	IGF inhibitor,[43] INSR inhibitor,[44] CYP inhibitor,[43] MEK inhibitor,[45] FAK inhibitor,[45] LCK inhibitor,[45] kinase inhibitor[45]
558441-90-0	anti-HIV,[46] GPCR ligand,[40,46] CXC chemokine receptor ligand[46]
Fluticasone furoate	nuclear hormone receptor ligand,[40,47] glucocorticoid receptor ligand,[40,47] anti-inflammatory,[48] anti-allergic[48]

Functional descriptors were initially chosen to represent well-documented specific protein targets (e.g., reverse transcriptase inhibitor, AKT inhibitor). Vague patents necessitated the use of broader functional descriptors including specific protein target classes (e.g., protease inhibitor, serotonergic) and downstream effects (e.g., analgesic, antiviral).

Database

The CheSS molecular embedding database was built from the SureChEMBL database to ensure high patent coverage for functional validation. This database was made achiral for compatibility with ChemBERTa, then duplicates and molecules with complexes were removed to reduce search redundancy.[26,51] For each molecule, the SMILES string was canonicalized using RDKit.[27] We reduced this dataset to exclude all SMILES strings that tokenized to more than 512 tokens, the maximum supported by ChemBERTa. This resulted in a database of 18,931,234 molecules. The database SMILES strings were embedded with ChemBERTa. The [CLS] token vector representations of the final layer were chosen to be the embeddings, as described in the original BERT paper.[52] These embeddings were then L2 normalized and stored in chunks of 100,000 SMILES string-embedding pairs for future cosine similarity calculations.

Canonicalization query types

ChemBERTa was trained on SMILES strings canonicalized using RDKit.[26,27] Different canonicalization algorithms result in different, but equally valid, standardized strings representing the same molecule, which we utilize to create three highly different queries for the same molecule. The first query type used RDKit with its default Python implementation settings. This algorithm was used to canonicalize the database and train the model. When converting molecules to SMILES, RDKit allows specification of which atom number to root the SMILES string to. The default is Atom 0, and each atom results in a different representation. The embedding cosine similarity was calculated between the default RDKit SMILES and the "Atom n" RDKit SMILES for each atom in the query molecule, as demonstrated in Figure S2. From these, we took the most dissimilar Atom n SMILES strings to be the second query type for each molecule (different atom depending on the specific molecule). To obtain a third dissimilar SMILES representation, OEChem 2.3.0, a markedly different canonicalization algorithm than RDKit, was used.[53] These SMILES strings were obtained from the PubChem website.

Similarity metrics

Various similarity metrics were used throughout, which include embedding cosine similarity, gestalt pattern matching similarity, fingerprint Tanimoto similarity, token vector length similarity, and token similarity.[54,55] Cosine similarity is a distance metric that calculates the angle between two vectors **A** and **B**:

$$\textit{Cosine Similarity} = \frac{\mathbf{A}\cdot\mathbf{B}}{\|\mathbf{A}\|\,\|\mathbf{B}\|}. \qquad \text{(Equation 1)}$$

A cosine similarity of 1 indicates the normalized vectors are the same, 0 means they are orthogonal to one another, and −1 means they are opposite of one another.

Gestalt pattern matching was chosen to calculate string similarity. This metric is calculated by dividing twice the number of matching characters (Km) by the total number of characters in both strings (S1, S2):

$$\textit{Gestalt Similarity} = \frac{2K_m}{|S_1|+|S_2|}. \qquad \text{(Equation 2)}$$

Matching characters are identified first from the longest common substring, with recursive counts in non-matching regions on both sides of the substring.

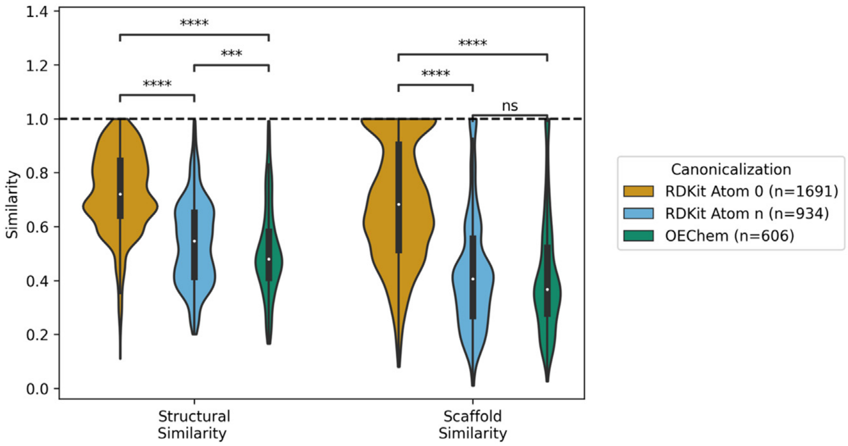

Figure 6. Structural and scaffold similarity between each query and its functionally similar search results

The functionally similar molecules, as determined from the LLM-assisted patent search, from the alternate canonicalization search results contain significantly more dissimilar structures and scaffolds compared to when the canonicalization is the same as the database. Structural similarity measured by whole-molecule fingerprint Tanimoto similarity; scaffold similarity measured by scaffold fingerprint Tanimoto similarity. Asterisks indicate the level of statistical significance for two-sided independent t tests (ns, $p < 1.0$; *$p < 0.05$; **$p < 0.01$; ***$p < 0.001$; ****$p < 0.0001$).

The metric ranges from a perfect match of 1 to a completely dissimilar string of 0. We used the difflib Python implementation of the gestalt pattern matching algorithm to calculate gestalt similarity. This is occasionally referred to in the manuscript as string similarity.

Fingerprint Tanimoto similarity was used to calculate the structural similarity between pairs of molecules. This method encodes substructures into a binary vector and then calculates the Tanimoto similarity between these encoded vectors. The Tanimoto/Jaccard similarity is the number of shared elements (intersection) between two sets A and B over the total number of unique elements in both sets (union) (Equation 3):

$$\text{Tanimoto Similarity} = \frac{A \cap B}{A \cup B}. \qquad \text{(Equation 3)}$$

This metric ranges from 1 (all elements shared) to 0 (no elements shared). The RDKit default implementation of fingerprint Tanimoto similarity was used herein. When fingerprint Tanimoto similarity was performed on the whole molecule, it was occasionally referred to in the paper as structural similarity, and when only performed on the scaffold it was referred to as scaffold similarity. Scaffolds were obtained using the Murcko scaffold method in RDKit.[27,28]

All SMILES were encoded into token vectors before being passed into the model. These tokenized vectors were used for additional comparisons to better understand search behavior. The first metric used from these was the ratio of token lengths between two vectors. The second metric was the token Tanimoto/Jaccard similarity (Equation 3) between the two molecules' token vectors and was used to determine the ratio of shared tokens between the two vectors. This metric ranges from 1 (all tokens shared) to 0 (no tokens shared).

LLM-assisted patent summarization and functional similarity determination

Chemical functionality was derived from the patent literature. To rapidly parse the patent literature to allow for a comprehensive analysis, a LLM-assisted patent summarization pipeline was developed. Using the PubChem database, achiral SMILES strings are converted to their corresponding same-connectivity PubChem CIDs (if available), which are then converted into the set of associated patents. To avoid excess LLM-associated API costs, a limit of 10 patent IDs per molecule was imposed, randomly selected from the set of linked patent IDs.

The patent IDs were then used as queries to obtain the patent title, abstract, and description from Google Scholar. The descriptions were capped at 3,500 characters to avoid token overflows and excess API costs. These were then appended to the following user prompt "Return a short set of three 1–3 word descriptors that best describe the chemical or pharmacological function(s) of the molecule described by the given patent title, abstract, and partial description (giving more weight to title and abstract). Be specific and concise, but not necessarily comprehensive (choose a small number of great descriptor). Follow the syntax '{descriptor_1}/{descriptor_2}/{etc}', writing 'NA' if nothing is provided. DO NOT BREAK THIS SYNTAX. The following is the patent":," and inputted into OpenAI's GPT-3.5-turbo (version accessed on July 15, 2023) with 0 temperature and the system prompt "You are an organic chemist summarizing chemical patents."[56] GPT-3.5-turbo was chosen over GPT-4 for the patent summarization step due to the high API cost per token for the latter model.[56,57]

The functional labels determined from GPT-3.5-turbo were converted into a set, passed into GPT-4 alongside the pre-determined query functional labels, and appended to the prompt "The following is a set of functional labels for two different molecules. Determine if the two molecules have similar functionality. If similar elements are found in both lists, these molecules have similar function. You must respond in the format '{yes or no} — {20 word maximum explanation}'" with 0 temperature and the system prompt "You are an organic chemistry expert."

A random 100 molecules from the 9,750 CheSS search results were used to validate the patent summarization and functional similarity determination pipeline. First, the functional labels were consolidated into a set with their associated patents to assess GPT-3.5-turbo's ability to summarize patents into quality functional descriptors. The authors read the 320 patents associated with the 100 random molecules and determined the following metrics for each label: label relevant to patent; label refers to molecule of interest (MOI), target of MOI, or downstream effects of MOI; label refers to MOI, target of MOI, downstream effects of MOI, or molecules of which MOI is an intermediate. These 100 molecules were then used to assess GPT-4's ability to determine functional similarity between the MOI and its associated CheSS query. The CheSS query descriptors were derived from the literature, aiming to be as specific as possible while taking into account the inherent vagueness of patents. For each molecule, the authors assessed GPT-4's success in determining functional similarity and the validity of its provided reasoning. Accuracy, precision, and recall were calculated for the former.

SUPPLEMENTAL INFORMATION

Supplemental information can be found online at https://doi.org/10.1016/j.patter.2023.100865.

ACKNOWLEDGMENTS

The authors acknowledge the Texas Advanced Computing Center and the Biomedical Research Computing Facility at The University of Texasat Austin for providing high-performance computing resources. We would also like to thank AMD for the donation of critical hardware and support resources from its HPC Fund. This work was supported by the Welch Foundation (F-1654), the NIH (R01GM146093), the Blumberg Centennial Professorship in Molecular Evolution, and the Reeder Centennial Fellowship in Systematic and Evolutionary Biology at The University of Texas at Austin. The authors would like to thank Charlie D. Johnson for useful criticism and discussion during the development of this project.

AUTHOR CONTRIBUTIONS

Conceptualization, C.K. and A.F.; methodology, C.K.; software, C.K. and A.F.; validation, C.K. and A.F; formal analysis, C.K.; investigation, C.K.;

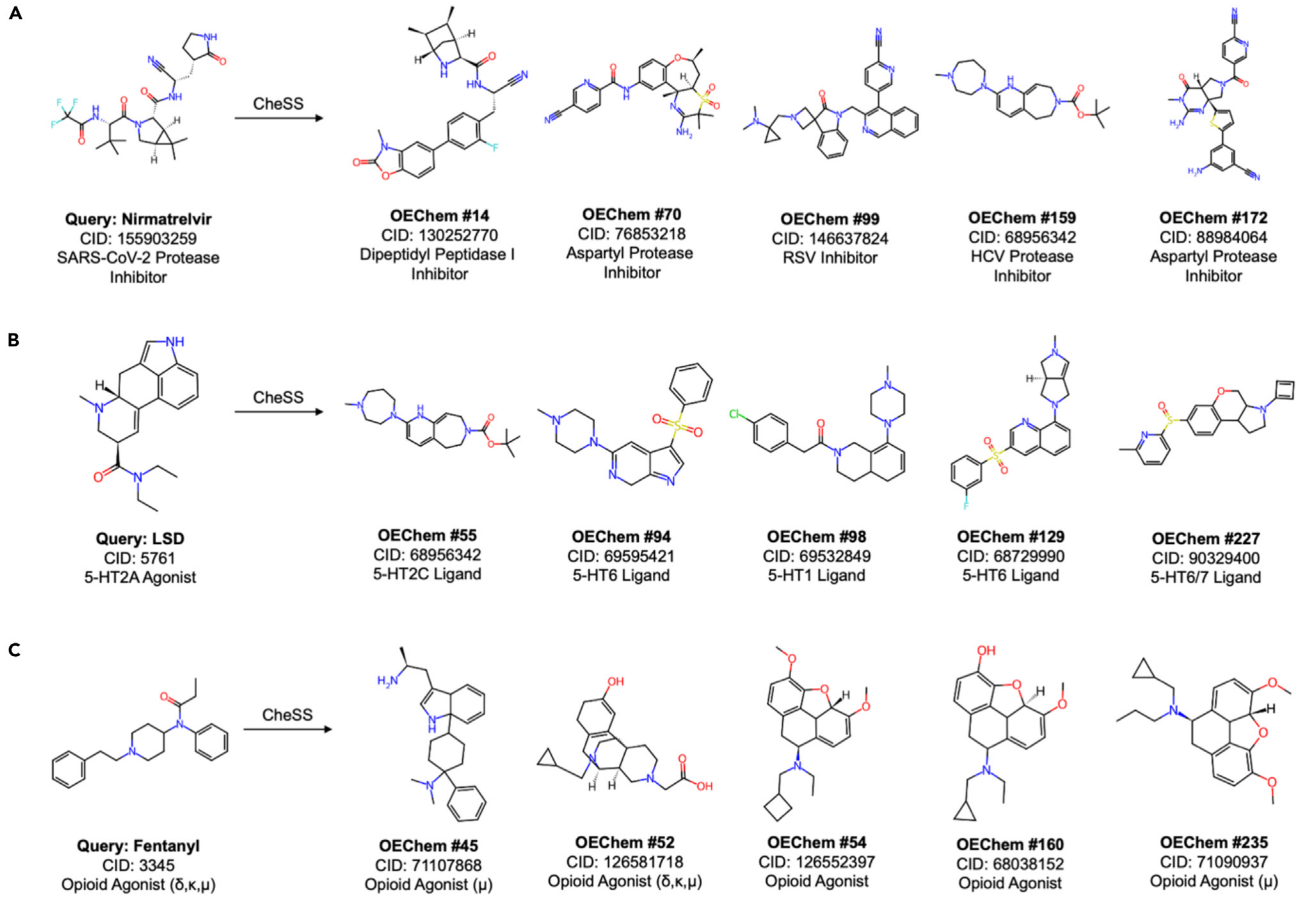

Figure 7. CheSS queries with alternative canonicalization identify functionally similar molecules with divergent structure
Results from the OEChem-canonicalized queries are shown, although RDKit Atom n also had structurally distinct functional analogues in the top 250 results.
(A) Nirmatrelvir, a peptidomimetic SARS-CoV-2 main protease inhibitor, returned a wide range of non-peptidomimetic protease and peptidase inhibitors.
(B) LSD, an ergoline 5-HT2A agonist, returned many non-ergoline 5-HT receptor ligands.
(C) Fentanyl, an elongated piperidine opioid agonist, returned several morphinan and non-fentanyl opiates.

resources, A.E. and C.W.; data curation, C.K. and A.F.; writing – original draft, C.K. and A.F.; writing – review and editing, C.K., A.F., A.E., and C.W.; visualization, C.K. and A.F.; supervision, A.E. and C.W.; funding acquisition, A.E. and C.W.

DECLARATION OF INTERESTS

A provisional patent application on the CheSS method of using alternative canonicalizations for querying a chemical language model has been filed. CheSS, along with associated code, has been made available through the GPL-2.0-only license.

DECLARATION OF GENERATIVE AI AND AI-ASSISTED TECHNOLOGIES IN THE WRITING PROCESS

During the preparation of this work the authors consulted OpenAI's ChatGPT to rephrase some complex sentences for increased clarity. After using this tool, the authors reviewed and edited the content as needed and take full responsibility for the content of the publication.

Received: May 17, 2023
Revised: August 9, 2023
Accepted: October 6, 2023
Published: October 30, 2023

REFERENCES

1. Li, Q., and Kang, C. (2020). Mechanisms of action for small molecules revealed by structural biology in drug discovery. Int. J. Mol. Sci. *21*, 5262.
2. Cragg, G.M., and Newman, D.J. (2005). Biodiversity: A continuing source of novel drug leads. Pure Appl. Chem. *77*, 7–24.
3. Fleming, A. (1941). Penicillin. Br. Med. J. *2*, 386.
4. Jiao, R.H., Xu, S., Liu, J.Y., Ge, H.M., Ding, H., Xu, C., Zhu, H.L., and Tan, R.X. (2006). Chaetominine, a cytotoxic alkaloid produced by endophytic Chaetomium sp. Org. Lett. *8*, 5709–5712.
5. Wani, M.C., and Horwitz, S.B. (2014). Nature as a Remarkable Chemist: A personal story of the discovery and development of Taxol. Anti Cancer Drugs *25*, 482–487.
6. Hughes, J.P., Rees, S., Kalindjian, S.B., and Philpott, K.L. (2011). Principles of early drug discovery. Br. J. Pharmacol. *162*, 1239–1249.
7. Martin, Y.C., Kofron, J.L., and Traphagen, L.M. (2002). Do structurally similar molecules have similar biological activity? J. Med. Chem. *45*, 4350–4358.
8. Pathan, H., and Williams, J. (2012). Basic opioid pharmacology: an update. Br. J. Pain *6*, 11–16.
9. Pu, L., Naderi, M., Liu, T., Wu, H.-C., Mukhopadhyay, S., and Brylinski, M. (2019). eToxPred: a machine learning-based approach to estimate the toxicity of drug candidates. BMC Pharmacol. Toxicol. *20*, 2–15.

CellPress
OPEN ACCESS

10. Yang, Y., Liu, M., and Kitchin, J.R. (2022). Neural network embeddings based similarity search method for atomistic systems. Dig. Dis. *1*, 636–644.
11. Lee, K., Jang, J., Seo, S., Lim, J., and Kim, W.Y. (2022). Drug-likeness scoring based on unsupervised learning. Chem. Sci. *13*, 554–565.
12. Sellner, M.S., Mahmoud, A.H., and Lill, M.A. (2023). Efficient virtual high-content screening using a distance-aware transformer model. J. Cheminf. *15*, 18.
13. Wei, B., Zhang, Y., and Gong, X. (2022). DeepLPI: a novel deep learning-based model for protein–ligand interaction prediction for drug repurposing. Sci. Rep. *12*, 18200.
14. Moret, M., Grisoni, F., Katzberger, P., and Schneider, G. (2022). Perplexity-based molecule ranking and bias estimation of chemical language models. J. Chem. Inf. Model. *62*, 1199–1206.
15. Moret, M., Pachon Angona, I., Cotos, L., Yan, S., Atz, K., Brunner, C., Baumgartner, M., Grisoni, F., and Schneider, G. (2023). Leveraging molecular structure and bioactivity with chemical language models for de novo drug design. Nat. Commun. *14*, 114.
16. Flam-Shepherd, D., Zhu, K., and Aspuru-Guzik, A. (2022). Language models can learn complex molecular distributions. Nat. Commun. *13*, 3293.
17. Weininger, D. (1988). SMILES, a chemical language and information system. 1. Introduction to methodology and encoding rules. J. Chem. Inf. Comput. Sci. *28*, 31–36.
18. Ross, J., Belgodere, B., Chenthamarakshan, V., Padhi, I., Mroueh, Y., and Das, P. (2022). Large-scale chemical language representations capture molecular structure and properties. Nat. Mach. Intell. *4*, 1256–1264.
19. Tingle, B.I., Tang, K.G., Castanon, M., Gutierrez, J.J., Khurelbaatar, M., Dandarchuluun, C., Moroz, Y.S., and Irwin, J.J. (2023). ZINC-22- A Free Multi-Billion-Scale Database of Tangible Compounds for Ligand Discovery. J. Chem. Inf. Model. *63*, 1166–1176.
20. Batool, M., Ahmad, B., and Choi, S. (2019). A structure-based drug discovery paradigm. Int. J. Mol. Sci. *20*, 2783.
21. Szilágyi, K., Flachner, B., Hajdú, I., Szaszkó, M., Dobi, K., Lőrincz, Z., Cseh, S., and Dormán, G. (2021). Rapid identification of potential drug candidates from multi-million compounds' repositories. combination of 2D similarity search with 3D ligand/structure based methods and in vitro screening. Molecules *26*, 5593.
22. Stumpfe, D., and Bajorath, J. (2011). Similarity searching. WIREs Comput. Mol. Sci. *1*, 260–282.
23. Gao, W., and Coley, C.W. (2020). The synthesizability of molecules proposed by generative models. J. Chem. Inf. Model. *60*, 5714–5723.
24. Arús-Pous, J., Johansson, S.V., Prykhodko, O., Bjerrum, E.J., Tyrchan, C., Reymond, J.-L., Chen, H., and Engkvist, O. (2019). Randomized SMILES strings improve the quality of molecular generative models. J. Cheminf. *11*, 1–13.
25. Mokaya, M., Imrie, F., van Hoorn, W.P., Kalisz, A., Bradley, A.R., and Deane, C.M. (2023). Testing the limits of SMILES-based de novo molecular generation with curriculum and deep reinforcement learning. Nat. Mach. Intell. *5*, 386–394.
26. Chithrananda, S., Grand, G., and Ramsundar, B. (2020). ChemBERTa: Large-Scale Self-Supervised Pretraining for Molecular Property Prediction. Preprint at arXiv. https://doi.org/10.48550/arXiv.2010.09885.
27. Landrum, G., and others. (2013). RDKit: A software suite for cheminformatics, computational chemistry, and predictive modeling. Greg Landrum *8*.
28. Bemis, G.W., and Murcko, M.A. (1996). The Properties of Known Drugs. 1. Molecular Frameworks. J. Med. Chem. *39*, 2887–2893. https://doi.org/10.1021/jm9602928.
29. Fischl, M.A., Richman, D.D., Hansen, N., Collier, A.C., Carey, J.T., Para, M.F., Hardy, W.D., Dolin, R., Powderly, W.G., Allan, J.D., et al. (1990). The safety and efficacy of zidovudine (AZT) in the treatment of subjects with mildly symptomatic human immunodeficiency virus type 1 (HIV) infection: a double-blind, placebo-controlled trial. Ann. Intern. Med. *112*, 727–737.
30. Shuang, S., and Jin, Q. (2009). Effecting of metal ions on the interaction between zidovudine and bovine serum albumin. Acta Phys. Chim. Sin. *25*, 1342–1346.
31. Timofeev, V.I., Zhukhlistova, N.E., and Kuranova, I.P. (2021). Molecular Dynamics Study of Escherichia coli Thymidine Phosphorylase in a Complex with 3'-Azidothymidine Inhibitor and Phosphate. Russ. J. Bioorg. Chem. *47*, 1231–1240. https://doi.org/10.1134/S1068162021060248.
32. Bush, K., and Bradford, P.A. (2016). β-Lactams and β-Lactamase Inhibitors: An Overview. Cold Spring Harb. Perspect. Med. *6*, a025247. https://doi.org/10.1101/cshperspect.a025247.
33. Akinosoglou, K., Schinas, G., and Gogos, C. (2022). Oral Antiviral Treatment for COVID-19: A Comprehensive Review on Nirmatrelvir/Ritonavir. Viruses *14*, 2540. https://doi.org/10.3390/v14112540.
34. Pieri, L., Pieri, M., and Haefely, W. (1974). LSD as an agonist of dopamine receptors in the striatum. Nature *252*, 586–588.
35. Titeler, M., Lyon, R.A., and Glennon, R.A. (1988). Radioligand binding evidence implicates the brain 5-HT 2 receptor as a site of action for LSD and phenylisopropylamine hallucinogens. Psychopharmacology (Berl.) *94*, 213–216.
36. Green, J.P., Weinstein, H., and Maayani, S. (1978). Defining the histamine H2-receptor in brain: the interaction with LSD. Quant. Struct. Act. Relatsh. Analg. Narc. Antagon. Hallucinog. *38*.
37. Ray, T.S. (2010). Psychedelics and the human receptorome. PLoS One *5*, e9019.
38. Armenian, P., Vo, K.T., Barr-Walker, J., and Lynch, K.L. (2018). Fentanyl, fentanyl analogs and novel synthetic opioids: a comprehensive review. Neuropharmacology *134*, 121–132.
39. Bagley, J.R., Thomas, S.A., Rudo, F.G., Spencer, H.K., Doorley, B.M., Ossipov, M.H., Jerussi, T.P., Benvenga, M.J., and Spaulding, T. (1991). New 1-(heterocyclylalkyl)-4-(propionanilido)-4-piperidinyl methyl ester and methylene methyl ether analgesics. J. Med. Chem. *34*, 827–841. https://doi.org/10.1021/jm00106a051.
40. Mysinger, M.M., Carchia, M., Irwin, J.J., and Shoichet, B.K. (2012). Directory of Useful Decoys, Enhanced (DUD-E): Better Ligands and Decoys for Better Benchmarking. J. Med. Chem. *55*, 6582–6594. https://doi.org/10.1021/jm300687e.
41. Heerding, D.A., Rhodes, N., Leber, J.D., Clark, T.J., Keenan, R.M., Lafrance, L.V., Li, M., Safonov, I.G., Takata, D.T., Venslavsky, J.W., et al. (2008). Identification of 4-(2-(4-Amino-1,2,5-oxadiazol-3-yl)-1-ethyl-7-{[(3 *S*)-3-piperidinylmethyl]oxy}-1 *H* -imidazo[4,5- *c*]pyridin-4-yl)-2-methyl-3-butyn-2-ol (GSK690693), a Novel Inhibitor of AKT Kinase. J. Med. Chem. *51*, 5663–5679. https://doi.org/10.1021/jm8004527.
42. Patel, P.R., Sun, H., Li, S.Q., Shen, M., Khan, J., Thomas, C.J., and Davis, M.I. (2013). Identification of potent Yes1 kinase inhibitors using a library screening approach. Bioorg. Med. Chem. Lett. *23*, 4398–4403. https://doi.org/10.1016/j.bmcl.2013.05.072.
43. Zimmermann, K., Wittman, M.D., Saulnier, M.G., Velaparthi, U., Langley, D.R., Sang, X., Frennesson, D., Carboni, J., Li, A., Greer, A., et al. (2008). Balancing oral exposure with Cyp3A4 inhibition in benzimidazole-based IGF-IR inhibitors. Bioorg. Med. Chem. Lett. *18*, 4075–4080. https://doi.org/10.1016/j.bmcl.2008.05.104.
44. Li, R., Pourpak, A., and Morris, S.W. (2009). Inhibition of the Insulin-like Growth Factor-1 Receptor (IGF1R) Tyrosine Kinase as a Novel Cancer Therapy Approach. J. Med. Chem. *52*, 4981–5004. https://doi.org/10.1021/jm9002395.
45. Wittman, M., Carboni, J., Attar, R., Balasubramanian, B., Balimane, P., Brassil, P., Beaulieu, F., Chang, C., Clarke, W., Dell, J., et al. (2005). Discovery of a 1 *H* -Benzoimidazol-2-yl)-1 *H* -pyridin-2-one (BMS-536924) Inhibitor of Insulin-like Growth Factor I Receptor Kinase with in Vivo Antitumor Activity. J. Med. Chem. *48*, 5639–5643. https://doi.org/10.1021/jm050392q.
46. Skerlj, R.T., Bridger, G.J., Kaller, A., McEachern, E.J., Crawford, J.B., Zhou, Y., Atsma, B., Langille, J., Nan, S., Veale, D., et al. (2010). Discovery of Novel Small Molecule Orally Bioavailable C−X−C

Chemokine Receptor 4 Antagonists That Are Potent Inhibitors of T-Tropic (X4) HIV-1 Replication. J. Med. Chem. *53*, 3376–3388. https://doi.org/10.1021/jm100073m.

47. Biggadike, K., Caivano, M., Clackers, M., Coe, D.M., Hardy, G.W., Humphreys, D., Jones, H.T., House, D., Miles-Williams, A., Skone, P.A., et al. (2009). Highly tractable, sub-nanomolar non-steroidal glucocorticoid receptor agonists. Bioorg. Med. Chem. Lett. *19*, 4846–4850. https://doi.org/10.1016/j.bmcl.2009.06.020.
48. Kaiser, H.B., Naclerio, R.M., Given, J., Toler, T.N., Ellsworth, A., and Philpot, E.E. (2007). Fluticasone furoate nasal spray: A single treatment option for the symptoms of seasonal allergic rhinitis. J. Allergy Clin. Immunol. *119*, 1430–1437. https://doi.org/10.1016/j.jaci.2007.02.022.
49. Urbina, F., Lentzos, F., Invernizzi, C., and Ekins, S. (2022). Dual use of artificial-intelligence-powered drug discovery. Nat. Mach. Intell. *4*, 189–191.
50. Kosonocky, C.W., Feller, A.L., Wilke, C.O., and Ellington, A.D. (2023). Alternative SMILES Representations for Chemical Similarity Vector Search Identifies Novel Functional Analogues. https://doi.org/10.5281/ZENODO.8226299.
51. Papadatos, G., Davies, M., Dedman, N., Chambers, J., Gaulton, A., Siddle, J., Koks, R., Irvine, S.A., Pettersson, J., Goncharoff, N., et al. (2016). SureChEMBL: a large-scale, chemically annotated patent document database. Nucleic Acids Res. *44*, D1220–D1228. https://doi.org/10.1093/nar/gkv1253.
52. Devlin, J., Chang, M.-W., Lee, K., and Toutanova, K. (2018). BERT: Pre-training of Deep Bidirectional Transformers for Language Understanding. Preprint at arXiv. https://doi.org/10.48550/arXiv.1810.04805.
53. OEChem, T. (2023). Version 2.3.0; (Santa Fe, NM: OpenEye Scientific Software).
54. Ratcliff, J.W., and Metzener, D.E. (1988). Pattern-matching-the gestalt approach. Dr. Dobb's J. *13*, 46.
55. Bajusz, D., Rácz, A., and Héberger, K. (2015). Why is Tanimoto index an appropriate choice for fingerprint-based similarity calculations? J. Cheminf. *7*, 1–13.
56. Brown, T., Mann, B., Ryder, N., Subbiah, M., Kaplan, J.D., Dhariwal, P., Neelakantan, A., Shyam, P., Sastry, G., Askell, A., et al. (2020). Language models are few-shot learners. Adv. Neural Inf. Process. Syst. *33*, 1877–1901.
57. OpenAI. (2023). GPT-4 Technical Report. Preprint at arXiv. https://doi.org/10.48550/ARXIV.2303.08774.

Patterns, Volume *4*

Supplemental information

Using alternative SMILES representations to identify novel functional analogues in chemical similarity vector searches

Clayton W. Kosonocky, Aaron L. Feller, Claus O. Wilke, and Andrew D. Ellington

Table S1. Different canonical SMILES string representations for each query molecule.

Query	Canon Alg.	SMILES
Zidovudine	RDKit Atom 0	Cc1cn(C2CC(N=[N+]=[N-])C(CO)O2)c(=O)[nH]c1=O
	RDKit Atom n	O=c1[nH]c(=O)c(C)cn1C1CC(N=[N+]=[N-])C(CO)O1
	OEChem	CC1=CN(C(=O)NC1=O)C2CC(C(O2)CO)N=[N+]=[N-]
Penicillin	RDKit Atom 0	CC1(C)SC2C(NC(=O)Cc3ccccc3)C(=O)N2C1C(=O)O
	RDKit Atom n	c1ccc(CC(=O)NC2C(=O)N3C2SC(C)(C)C3C(=O)O)cc1
	OEChem	CC1(C(N2C(S1)C(C2=O)NC(=O)CC3=CC=CC=C3)C(=O)O)C
Nirmatrelvir	RDKit Atom 0	CC(C)(C)C(NC(=O)C(F)(F)F)C(=O)N1CC2C(C1C(=O)NC(C#N<br>)CC1CCNC1=O)C2(C)C
	RDKit Atom n	N(C(=O)C1C2C(CN1C(=O)C(NC(=O)C(F)(F)F)C(C)(C)C)C2(<br>C)C)C(C#N)CC1CCNC1=O
	OEChem	CC1(C2C1C(N(C2)C(=O)C(C(C)(C)C)NC(=O)C(F)(F)F)C(=O<br>)NC(CC3CCNC3=O)C#N)C
LSD	RDKit Atom 0	CCN(CC)C(=O)C1C=C2c3cccc4[nH]cc(c34)CC2N(C)C1
	RDKit Atom n	c1ccc2[nH]cc3c2c1C1=CC(C(=O)N(CC)CC)CN(C)C1C3
	OEChem	CCN(CC)C(=O)C1CN(C2CC3=CNC4=CC=CC(=C34)C2=C1)C
Fentanyl	RDKit Atom 0	CCC(=O)N(c1ccccc1)C1CCN(CCc2ccccc2)CC1
	RDKit Atom n	c1(CCN2CCC(N(C(=O)CC)c3ccccc3)CC2)ccccc1
	OEChem	CCC(=O)N(C1CCN(CC1)CCC2=CC=CC=C2)C3=CC=CC=C3
SB-759335-B	RDKit Atom 0	CCn1c(-c2nonc2N)nc2cncc(C(=O)N3CCNCC3)c21
	RDKit Atom n	c1ncc2nc(-c3nonc3N)n(CC)c2c1C(=O)N1CCNCC1
	OEChem	CCN1C2=C(C=NC=C2C(=O)N3CCNCC3)N=C1C4=NON=C4N
BMS-536924	RDKit Atom 0	Cc1cc(N2CCOCC2)cc2[nH]c(-c3c(NCC(O)c4cccc(Cl)c4)cc<br>[nH]c3=O)nc12
	RDKit Atom n	C(Nc1cc[nH]c(=O)c1-c1nc2c(C)cc(N3CCOCC3)cc2[nH]1)C<br>(O)c1cccc(Cl)c1
	OEChem	CC1=CC(=CC2=C1N=C(N2)C3=C(C=CNC3=O)NCC(C4=CC(=CC=C<br>4)Cl)O)N5CCOCC5
558441-90-0	RDKit Atom 0	NCCCCN(Cc1nc2ccccc2[nH]1)C1CCCc2cccnc21
	RDKit Atom n	c1cccc2[nH]c(CN(CCCCN)C3CCCc4cccnc43)nc12
	OEChem	C1CC(C2=C(C1)C=CC=N2)N(CCCCN)CC3=NC4=CC=CC=C4N3
Fluticasone furoate	RDKit Atom 0	CC1CC2C3CC(F)C4=CC(=O)C=CC4(C)C3(F)C(O)CC2(C)C1(OC<br>(=O)c1ccco1)C(=O)SCF
	RDKit Atom n	o1cccc1C(=O)OC1(C(=O)SCF)C(C)CC2C3CC(F)C4=CC(=O)C=<br>CC4(C)C3(F)C(O)CC21C
	OEChem	CC1CC2C3CC(C4=CC(=O)C=CC4(C3(C(CC2(C1(C(=O)SCF)OC(<br>=O)C5=CC=CO5)C)O)F)C)F

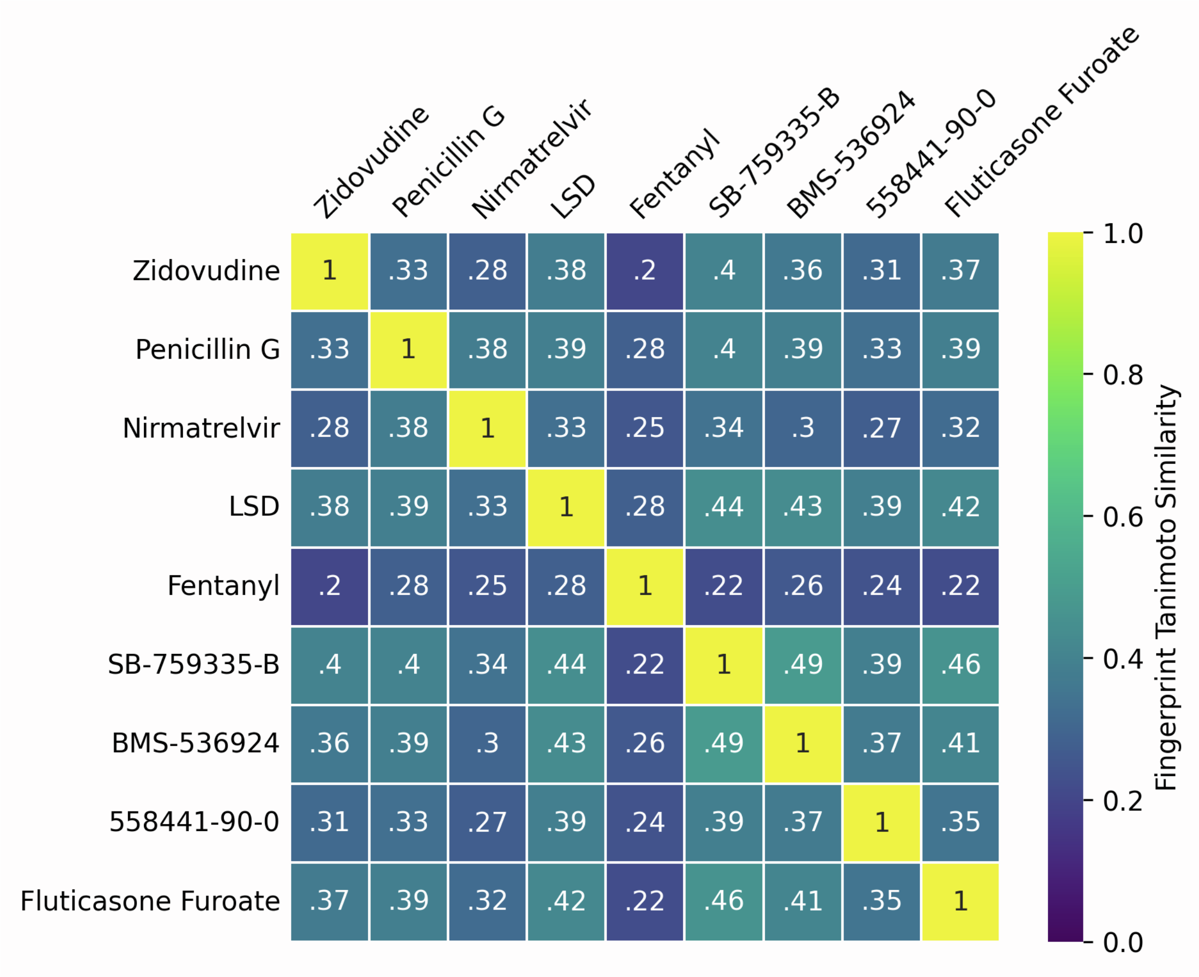

Figure S1: Fingerprint Tanimoto coefficients between each of the query molecules.

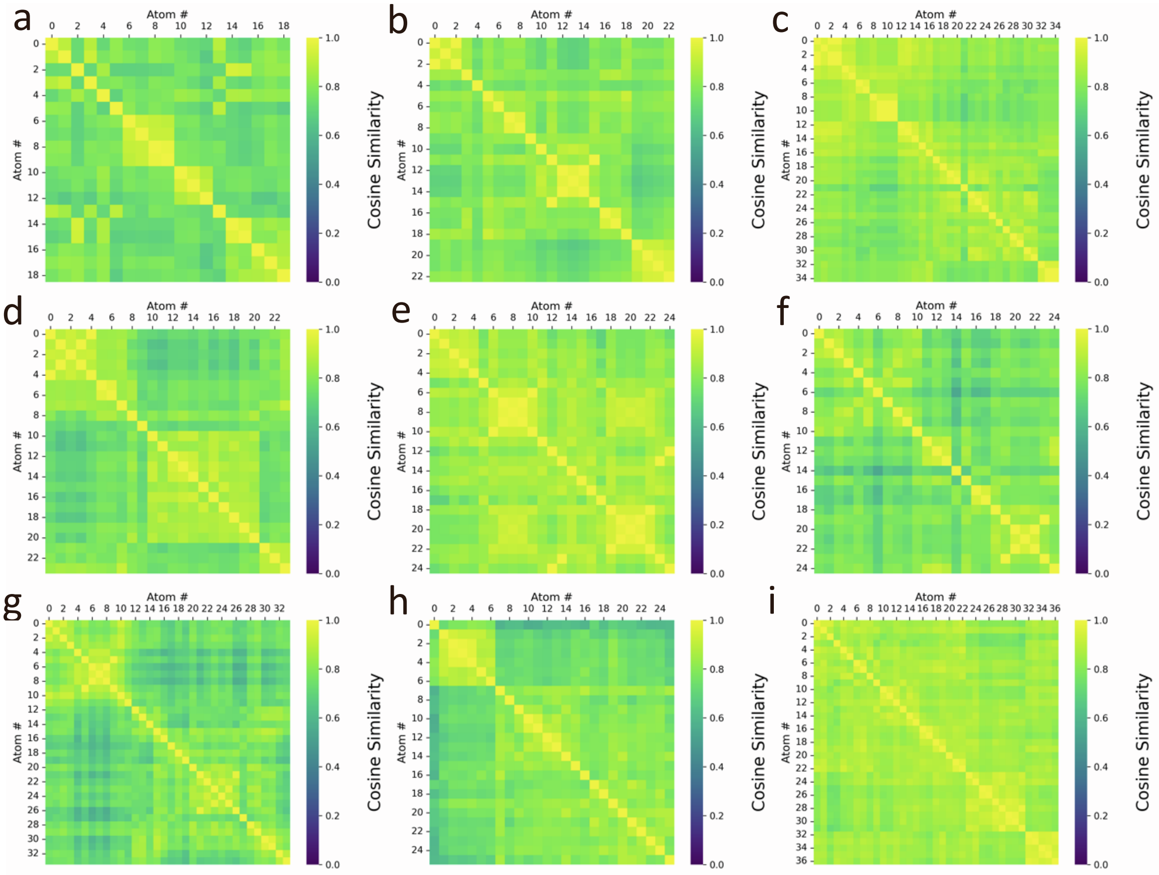

Figure S2: Feature cosine similarity of each RDKit canonicalized query depending on the chosen root atom number. (a-h). In order: zidovudine, penicillin, nirmatrelvir, LSD, fentanyl, SB-759335-B, BMS-536924, 558441-90-0, fluticasone furoate. The canonicalized variant with the lowest feature cosine similarity to the Atom 0 representation was chosen as the “RDKit Atom n” query. The root atoms providing most dissimilar feature vectors were 15 for zidovudine, 13 for penicillin, 21 for nirmatrelvir, 11 for LSD, 17 for fentanyl, 14 for SB-759335-B, 17 for BMS-536924, 10 for 558441-90-0, and 31 for fluticasone furoate.

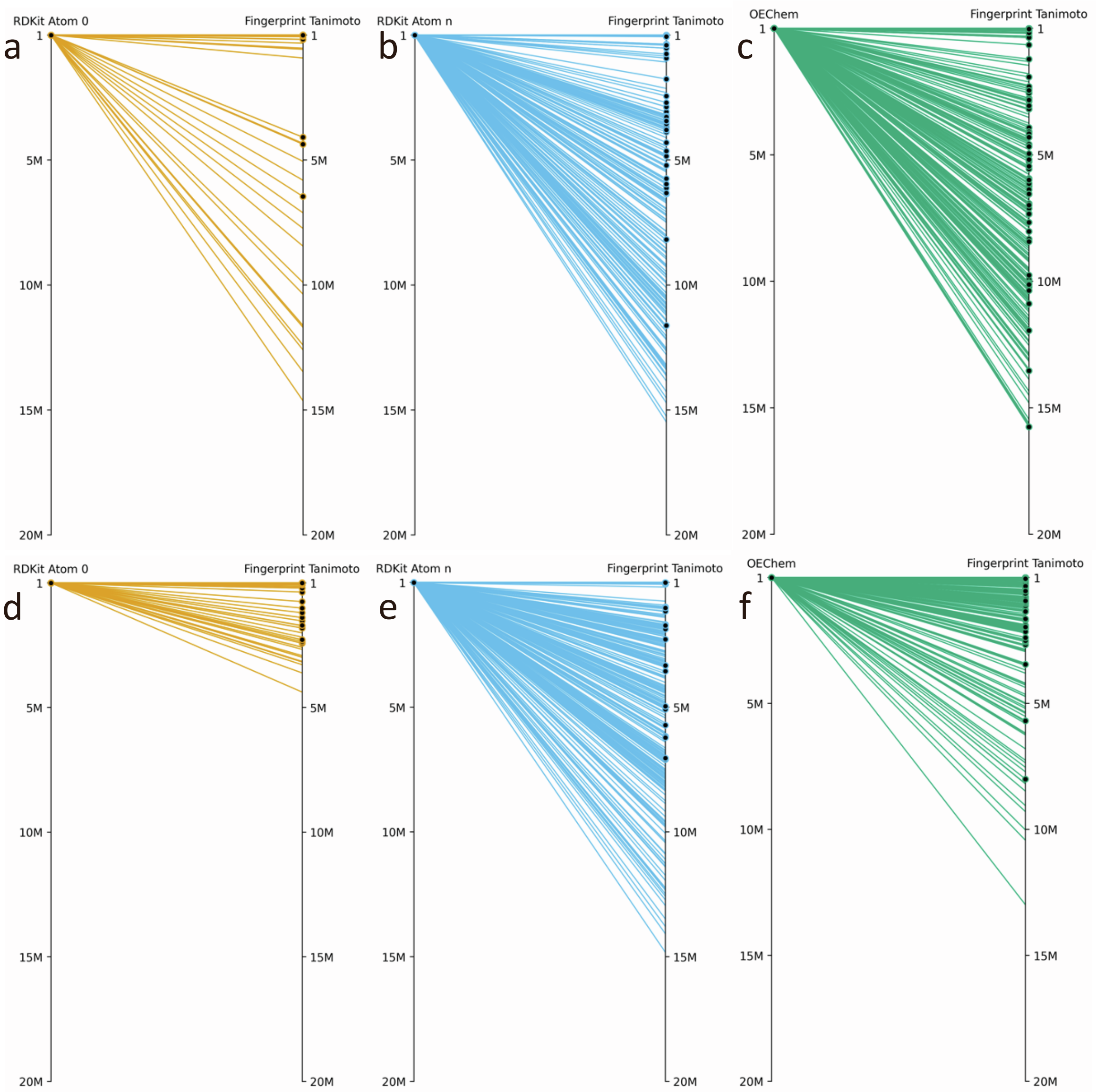

Figure S3. The index rank of each canonicalization's top 250 results for zidovudine and penicillin compared to the index rank that these same molecules scored in a fingerprint Tanimoto search. Black dot indicates molecules functionally similar to the query, as determined by the GPT-assisted patent search. **(a-c)** Zidovudine. **(d-f)** Penicillin.

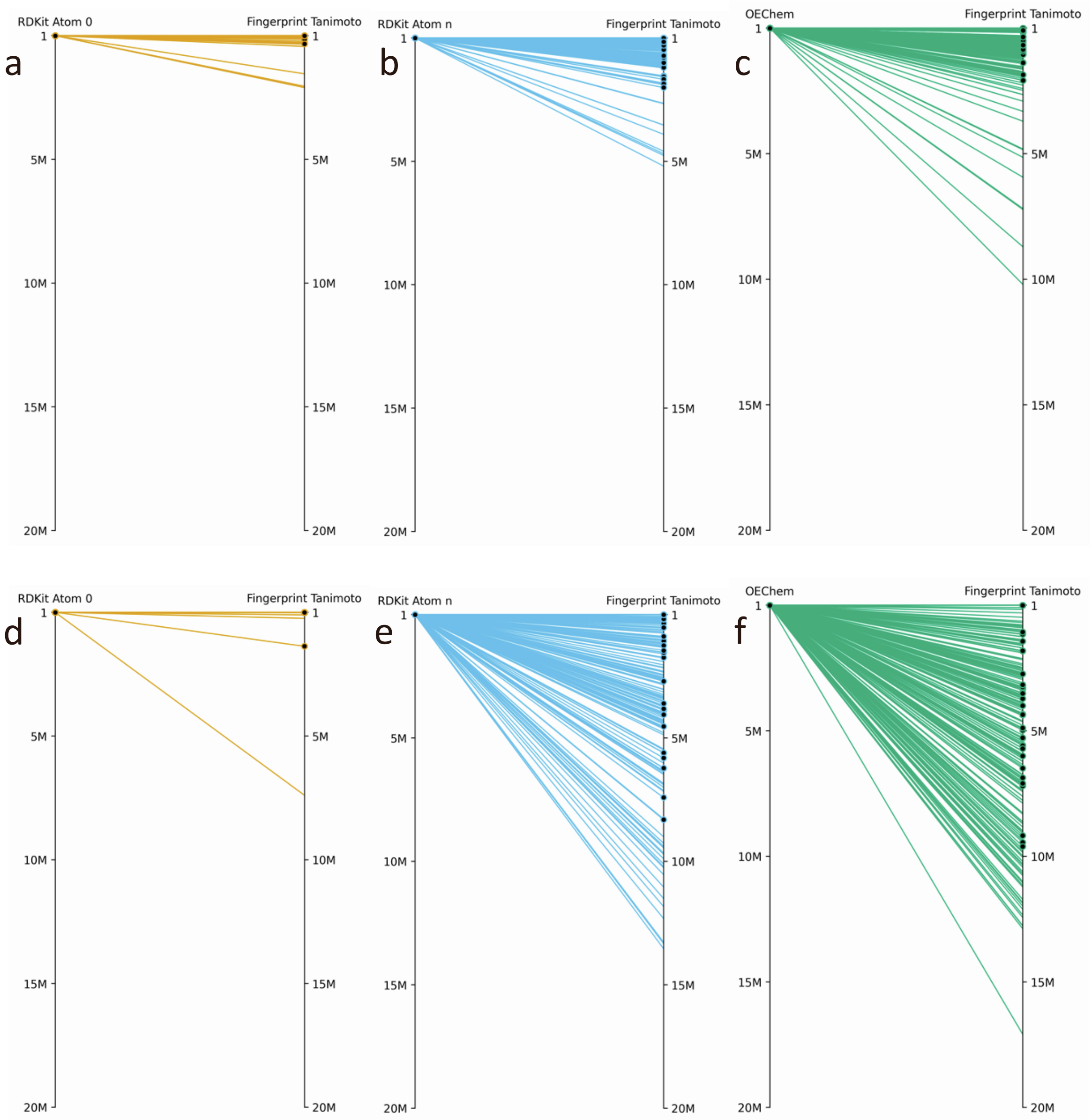

Figure S4. The index rank of each canonicalization's top 250 results for nirmatrelvir and LSD compared to the index rank that these same molecules scored in a fingerprint Tanimoto search. Black dot indicates molecules functionally similar to the query, as determined by the GPT-assisted patent search. **(a-c)** Nirmatrelvir. **(d-f)** LSD.

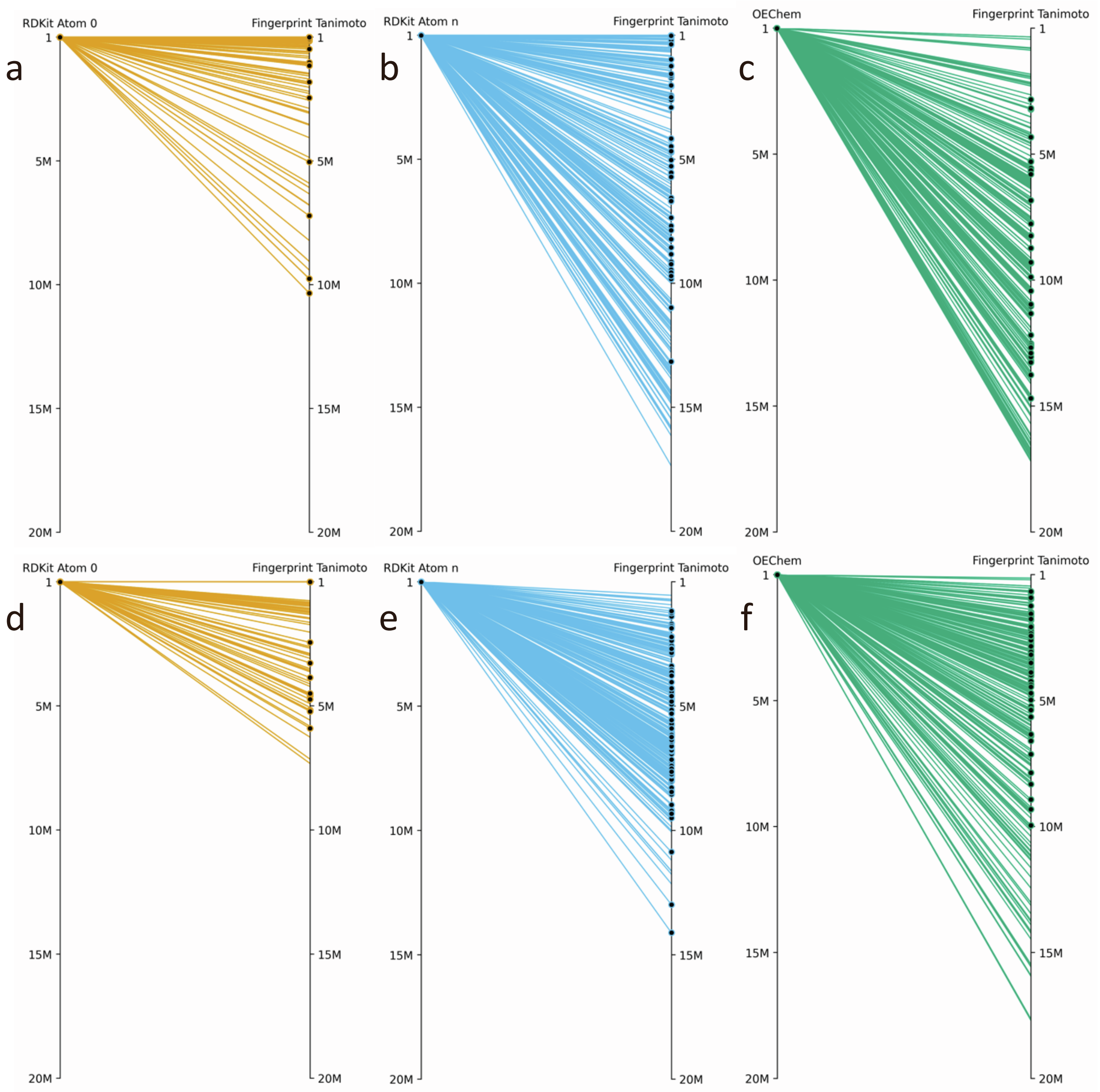

Figure S5. The index rank of each canonicalization's top 250 results for fentanyl and SB-759335-B compared to the index rank that these same molecules scored in a fingerprint Tanimoto search. Black dot indicates molecules functionally similar to the query, as determined by the GPT-assisted patent search. **(a-c)** Fentanyl. **(d-f)** SB-759335-B.

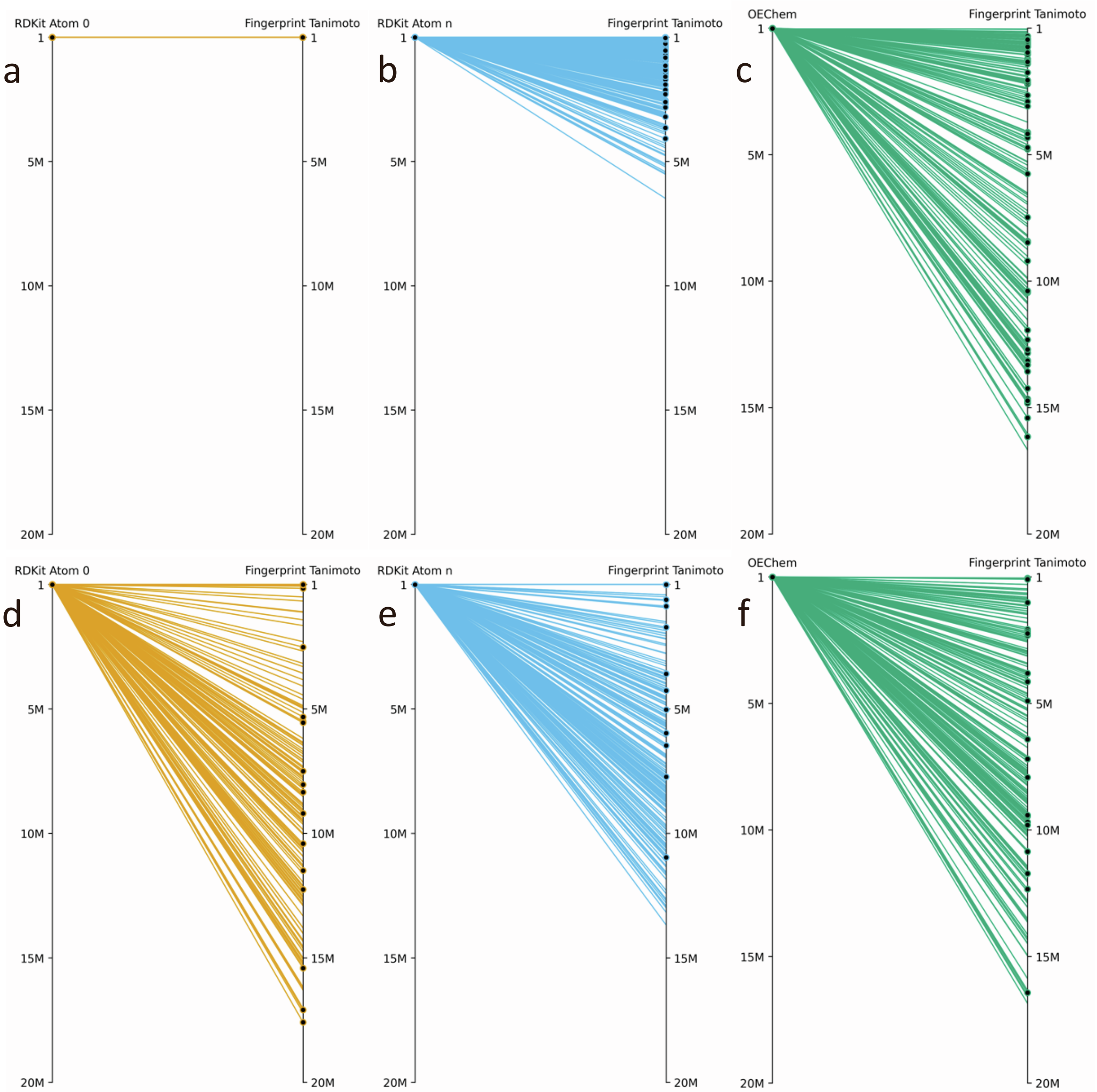

Figure S6. The index rank of each canonicalization's top 250 results for BMS-536924 and 558441-90-0 compared to the index rank that these same molecules scored in a fingerprint Tanimoto search. Black dot indicates molecules functionally similar to the query, as determined by the GPT-assisted patent search. **(a-c)** BMS-536924. **(d-f)** 558441-90-0.

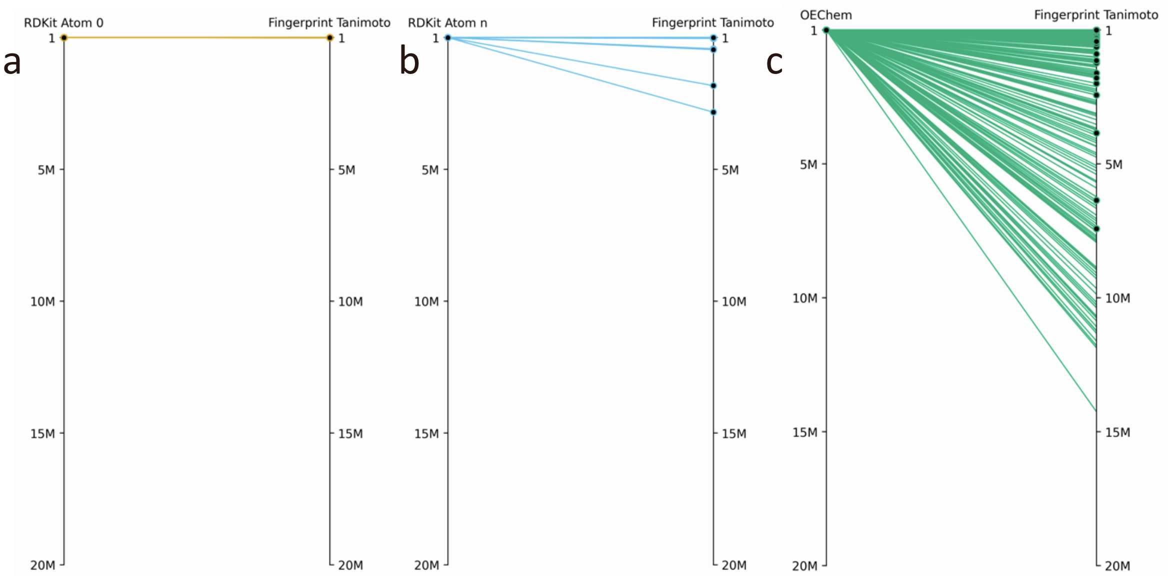

Figure S7. The index rank of each canonicalization's top 250 results for fluticasone furoate compared to the index rank that these same molecules scored in a fingerprint Tanimoto search. Black dot indicates molecules functionally similar to the query, as determined by the GPT-assisted patent search. **(a-c)** Fluticasone furoate.
